# Influence of the Abiotic Stress Conditions, Waterlogging and Drought, on the Bitter Sensometabolome as Well as Agronomical Traits of Six Genotypes of *Daucus carota*

**DOI:** 10.3390/foods10071607

**Published:** 2021-07-11

**Authors:** Christian Schmid, Sapna Sharma, Timo D. Stark, Daniela Günzkofer, Thomas F. Hofmann, Detlef Ulrich, Frank Dunemann, Thomas Nothnagel, Corinna Dawid

**Affiliations:** 1 Food Chemistry and Molecular Sensory Science, Technical University of Munich, Lise-Meitner-Str. 34, 85354 Freising, Germany; chris.schmid@tum.de (C.S.); sapna.sharma@tum.de (S.S.); timo.stark@tum.de (T.D.S.); daniela.guenzkofer@freenet.de (D.G.); thomas.hofmann@tum.de (T.F.H.); 2 Julius Kühn-Institut (JKI), Federal Research Centre for Cultivated Plants, Institute for Ecological Chemistry, Plant Analysis and Stored Product Protection, Königin-Luise-Straße 19, 14195 Berlin, Germany; detlef.ulrich@web.de; 3 Julius Kühn-Institut (JKI), Federal Research Centre for Cultivated Plants, Institute for Breeding Research on Horticultural Crops, Erwin-Baur-Strasse 27, 06484 Quedlinburg, Germany; frank.dunemann@julius-kuehn.de (F.D.); thomas.nothnagel@julius-kuehn.de (T.N.)

**Keywords:** UHPLC-MS/MS, bitter off-taste compounds, 6-methoxymellein, vaginatin, falcarindiol, polyacetylenes, carrots, *Daucus carota*

## Abstract

Cultivated carrot is one of the most important vegetable plants in the world and favored by consumers for its typically sweet flavor. Unfortunately, the attractive sensory quality is hindered by a sporadic bitter off-taste. To evaluate the influence of the abiotic stress conditions, waterlogging and drought, on the bitter sensometabolome as well as agronomical traits of six genotypes of *Daucus carota*, a field trial was performed. Enabling the accurate tracing of carrots’ bitter compounds and therefore their metabolic changes, a fast and robust high-throughput UHPLC-MS/MS was developed and validated. Remarkably, the genotypes are the driving source for the biological fate of the bitter metabolites that are reflected in concentrations, dose-over-threshold factors, and fold changes. A certain influence of the irrigation level is observable but is overruled by its cultivar. Therefore, metabolic stress response in carrots seems to be genotype dependent. Hence, this study might help to plant specific carrot genotypes that are adapted to stress conditions evoked by future climatic changes.

## 1. Introduction

Cultivated carrot (*Daucus carota* ssp. *sativus Hoffm*.), either consumed as a fresh or processed product, is one of the most important vegetable plants in the world [1]. Because of its high yield potential with a current annual world production of nearly 40 million tons and a total growing area of more than 1.1 million ha in 2018, the carrot ranks among the top ten vegetable crops [2].

**Figure 1:**
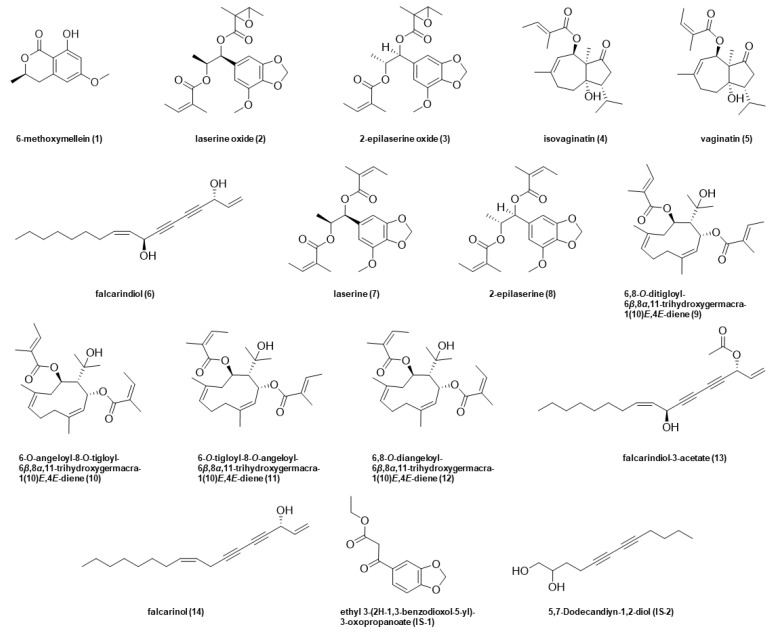
Chemical structure of known bitter compounds **1**–**14** in carrots and internal standards **1** and **2**.

Consumers favor carrots for their typically sweet notes inter alia. Unfortunately, the attractive sensory quality is hindered by a sporadic bitter off-taste, which is often the reason for consumer complaints and, therefore, a major problem for vegetable processors. Known bitter off-taste compounds in carrots are 6-methoxymellein (**1**), laserine oxide (**2**), 2-epilaserine oxide (**3**), isovaginatin (**4**), vaginatin (**5**), falcarindiol (**6**), laserine (**7**), 2-epilaserine (**8**), 6,8-*O*-ditigloyl-(**9**), 6-*O*-angeloyl-,8-*O*-tigloyl-(**10**), 6-*O*-tigloyl-, 8-*O*-angeloyl-(**11**), 6-,8-*O*-diangeloyl-6*β*,8α,11-trihydroxygermacra-1(10)*E*,4*E*-diene (**12**), falcarindiol-3-acetate (**13**), and falcarinol (**14**) (Figure 1) [3,4,5,6]. This bitter off-flavor could be evoked by abiotic (e.g., drought, water, salt) and biotic stress (e.g., *Trioza apicalis* Förster) factors during growing period as well as harvesting, transportation, storage, and processing [7,8,9,10,11,12,13]. Facing climate change and, consequently, more and more extreme weather periods, like droughts and water bottleneck, saline grounds, or sudden waterloggings, it is crucial to get a better understanding of carrots’ metabolome and tastants alteration in response to different stress conditions.

Although several biotic and abiotic stress factors during growth in the field as well as during post-harvest storage were reported to influence the amounts of secondary metabolites in carrots, such as the key bitter tastants present in native carrots, the C_17_-polycetylenes [14,15,16], and Matthias Wüst already highlighted in his perspective that abiotic and biotic stress challenges influence odor and taste stimuli concentrations and profiles in crop plants [17], encompassing knowledge on the fate of all carrot bitter compounds (**1**–**14**) under water stress conditions is rather limited and unclear. So far, only the effect of water stress on the polyacetylenic constituents of carrots (*D. carota cv Orlando Gold*) was investigated in plants grown in the greenhouse under normal conditions (ns) and under conditions simulating drought (dr) or waterlogging (wl). Eleven compounds were isolated among the carrot polyacetylenes falcarinol, falcarindiol, and falcarindiol-3-monoacetate. Three of the eight unidentified compounds were produced only by one or both stressed samples; the other eight, including the three known polyacetylenes, were less concentrated in the stressed samples than in the control [16]. For those falcarinol-type polyacetylenes, a wide range of bioactivities have been reported, including allergenic, antibacterial, antimycobacterial, antifungal, anti-cancer, and anti-inflammatory activities as well as bitter taste [3,4,5,6,18].

A series of attempts have been undertaken in recent years to quantitatively determine the contents of polyacetylenes, especially falcarindiol (**2**), falcarinol (**3**), and falcarindiol-3-acetate (**4**), in *Daucus carota*. The majority of polyacetylene analyses were based on the use of HPLC-UV [19,20] and gas-chromatographic techniques (GC–FID and/or GC–MS) [9,21,22,23] (for review, see [24]). The combination of HPLC and mass spectrometry (LC–MS/MS) has only recently been successfully applied to the analysis of polyacetylenes in carrots and in biofluidics obtained in a rat intervention study [24]. Although the analysis of polyacetylens via LC-MS/MS nowadays is state-of-the art, a unified quantitation method to characterize the bitter flavor of carrots and the impact of stress conditions to its up-regulation is still missing.

In order to gain a more comprehensive knowledge on the chemical mechanisms involved in the bitter sensometabome changes of cultivated carrots in response to the abiotic stress factors of waterlogging and drought, six different stressed and non-stressed carrot genotypes in field were comparatively screened via LC-MS/MS. Revealing the presence of the corresponding metabolites, a fast and robust high-throughput UHPLC-MS/MS_MRM_ was developed and validated to highlight metabolic changes tracing bitter taste depending on genotype and/or stress condition.

## 2. Materials and Methods

Chemicals. The following compounds were commercially obtained from the sources given in parentheses: ethylacetate (VWR International GmbH, Darmstadt, Germany); ethyl 3-(2H-1,3-benzodioxol-5-yl)-3-oxopropanoate and 5,7-dodecandiyn-1,2-diol used as internal standards (**IS-1**, **-2**) (abcr GmbH, Karlsruhe, Germany); methanol HPLC grade (J. T. Baker, Neventor, The Netherlands); formic acid and all deuterated solvents were obtained from Sigma-Aldrich (Taufkirchen, Germany); solvents used for UPLC-MS/MS analysis were of LC-MS grade (Honeywell, Seelze, Germany). Ultrapure water for LC-MS analysis was purified by means of a Milli-Q water advantage A 10 water system (Millipore, Molsheim, France). Compounds **1**–**5** and **7**–**13** (Figure 1) were used as reference compounds and were isolated from *Daucus carota* L. as reported earlier [5]. Bisacetylenic oxylipins falcarindiol (**6**) and falcarinol (**14**) were purchased from Carbosynth Limited (Compton, Berkshire, United Kingdom).

Sample Material. Seeds of six orange carrot (*Daucus carota* spp. *sativus*) cultivars obtained from various collections (Figure 1 and Table 1) were manually sown in the soil-seedbed under a plastic tunnel (rain-out-shelter). The experiment was designed as randomized block trial with each three irrigation variants and each three biological replications. Each plot was a double row with 30 cm distance and 1 m length. The plots were drip irrigated by using a sprinkler hose system (Supplementary Figure S1).

During the juvenile plant phase, 35 days from seed germination to six leaf stage, plants were six times drop irrigated together 55.5 L/m (each row). After that, the plots of the three blocks were irrigated for conditions waterlogging (wl): daily, approximately 5.0 L/m; for control (non-stressed (ns)): each five days, 5 L/m; and for drought (dr): each 10 days, 2.5 L/m; in summary, over the season: 260, 100, 60 L/m, respectively.

The plants were fertilized every three weeks using 200 mL of a 0.3% commercial NPK-fertilizer solution WUXAL^®^Super (Wilhelm Haug GmbH & Co.KG, Düsseldorf, Germany) applied per row and neither fungicides nor pesticides applied during the cultivation. Plants were harvested 112 days after sowing. First the aerial plant high (PH; cm) and the total leaf mass per plot (LM; g) were measured. Then, the number (n) and total weight (m) was estimated of the marketable root per plot (MR), cracked and diseased roots (DR), and undeveloped roots (UR). The agronomic data were estimated with the ANOVA and Tukey HSD procedure using the Statistica 7.1 software (StatSoft, Hamburg, Germany). Each of 10 representative healthy and marketable roots per plot and application were subsequently frozen until analyses.

Quantitation of carrot bitter compounds. Sample Preparation. Thawed carrot samples were cut into small pieces, shock frosted in liquid nitrogen (Westfalen AG, Münster, Germany), and finely powdered (Retsch Grindomix GM200, Retsch, Haan, Germany) for 1 min at 10,000 rpm. Then, 30 g carrot sample was macerated with 100 mL EtOAc for 2 min (Ultra Turrax T 25 digital, IKA-Werke, Staufen im Breisgau, Germany), followed by filtration and repeated extraction/filtration (twice). Combined filtrates were concentrated under reduced pressure at 40 °C and lyophilized to dryness. Until mass spectrometric investigation, samples were stored at −80 °C and then reconstituted with 80/20 ACN/H_2_O (*v*/*v*) to a concentration of 1 mg/mL in an ultra-sonic bath (Bandelin Sonorex Super RK 510 H (Bandelin, Berlin, Germany). Samples (200 µL) were mixed with internal standard (IS) solution (10 µL) to a concentration of 0.0055 µmol/L (**IS-1**) and 0.03 µmol/L (**IS-2**), equilibrated for 3 min via vortexing, and subjected to LC-MS/MS analysis.

Calibration. A stock solution of ethyl 3-(2H-1,3-benzodioxol-5-yl)-3-oxopropanoate (**IS-1**, 11.5 µmol/L) and 5,7-dodecandiyn-1,2-diol (**IS-2**, 64.8 µmol/L) as well as the analytes **1**–**14** were prepared in MeOD (**1**–**5**, **7**–**12**, **IS-1**) or CDCl_3_ (**6**, **13**, **14**, **IS-2**), and its exact concentration was verified by means of quantitative NMR (qNMR), respectively. Thereafter, different analyte concentrations (Supplementary Table S1) were mixed with constant concentrations of IS (**IS-1**, 0.0055 µmol/L; **IS-2**, 0.03 µmol/L). Triplicate UHPLC-MS/MS analysis calibration curves were prepared by plotting peak area ratios of analytes (**1**–**5**, **7**–**12** or **6**, **13**, **14**) to the internal standard **IS-1** or **IS-2** against concentration ratios of respective analytes to internal standard using linear regression (MultiQuant 3.0.1, Sciex, Darmstadt, Germany), giving equations as stated in Supplementary Table S2. The responses were linear, with a correlation coefficient of >0.98 for chosen molar ratios, and the concentrations of the analytes **1**–**14** in the samples were calculated using the respective calibration functions. Lowest concentration used for calibration of the analytes **1**–**14** was, in all cases, above the signal-to-noise ratio of 10 (Table S2).

Recovery. In order to determine the recovery rate of each compound in *Daucus carota*, fresh carrots from a local supermarket were extracted as stated above. Extracted samples were spiked with four defined concentration levels of each analyte **1**–**14** (Table 1) as well as fixed concentrations of the internal standards (**IS-1**, **IS-2**). In addition, control samples containing only internal standard were analyzed. After sample preparation, the sensometabolites were quantified in triplicates by means of UPLC-MS/MS to give mean recovery rates as follows: **1** (79.7%), **2** (96.2%), **3** (98.9%), **4** (83.3%), **5** (92.7%), **6** (105.6%), **7** (115.3%), **8** (98.6%), **9** (99.6%), **10** (96.7%), **11** (88.0%), **12** (85.2%), **13** (119.7%), and **14** (103.0%).

Precision. For the intraday precision, five aliquots of the same carrot extract were analyzed on the same day. The precision of the developed method was determined in triplicate analysis and expressed by the relative standard deviation given in parentheses: **1** (2.3%), **2** (4.4%), **3** (3.1%), **4** (2.1%), **5** (2.0%), **6** (1.6%), **7** (13.5%), **8** (2.9%), **9** (3.0%), **10** (3.7%), **11** (3.8%), **12** (5.0%), **13** (2.0%), and **14** (2.1%). The interday precision of the method was determined by triplicate analysis on three consecutive days of five aliquots of the same batch of carrots and expressed by the relative standard deviation given in parentheses: **1** (1.6%), **2** (3.4%), **3** (3.4%), **4** (3.2%), **5** (2.7%), **6** (4.2%), **7** (15.3%), **8** (1.5%), **9** (5.0%), **10** (9.6%), **11** (5.7%), **12** (10.7%), **13** (6.8%), and **14** (5.4%).

Quantitative Nuclear Magnetic Resonance Spectroscopy (qNMR). For determination of calibration compound concentration, each substance was solved in acetonitrile-d_4_/D_2_O (70/30, *v*/*v*). ^1^H-NMR spectra were acquired at 298 K using an Avance III 400 MHz spectrometer (Bruker, Rheinstetten, Germany) equipped with a Bruker 5 mm BBI probe and TopSpin 3.2 for data acquisition and processing. Aliquots (600 µL) of each stock solution were analyzed in 5 mm × 7 in. NMR tubes (Bruker, Faellanden, Switzerland). In order to get quantitative information for ^1^H-NMR experiments, Bruker pulse program was utilized, and to ensure good-quality spectra, the NMR probe was manually tuned and matched to 50 Ω resistive impedance to minimize RF reflection followed by automatic optimization of the lock phase, shimming of the sample (z^1^—z^5^, xyz, z^1^—z^5^), and individual determination of the 90° pulse using the AU program “pulsecal sn” (Bruker Topspin 3.2). All spectra were recorded with settings reported previously [25].

**Figure 2:**
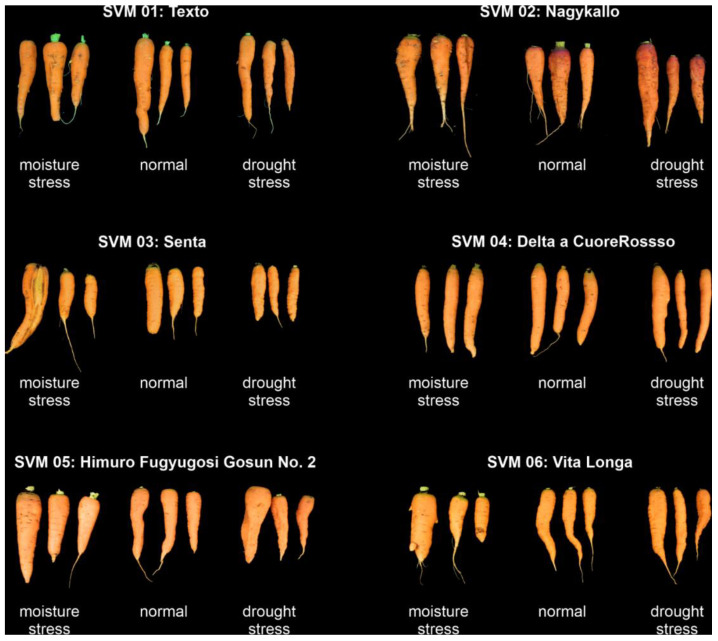
Roots’ expression of water-stressed carrot cultivars using the rain-out-shelter experiment.

Ultra-High-Performance Liquid Chromatography-Mass Spectrometry (UHPLC-MS/MS). Quantitative analyses of carrot samples were performed on a Shimadzu Nexera X2 UHPLC system (Shimadzu Germany, Duisburg, Germany) consisting of two binary pumps (LC30AD), auto sampler (SIL30AC), a column oven (CTO30A), and a controller (CBM20A), which is connected to QTrap 6500 triple quadrupole mass spectrometer (Sciex, Darmstadt, Germany). For data acquisition, Analyst 1.6.3 software (Sciex, Darmstadt, Germany) was used. Aliquots (1 µL) of sample extracts, which were stored until analysis in autosampler at 4 °C, were chromatographed on an analytical Kinetex 1.7 µm, C18 100 Å, 100 mm × 2.1 mm column (Phenomenex, Aschaffenburg, Germany) at 40 °C column temperature using acetonitrile (0.1% formic acid, *v*/*v*) as solvent B and water (0.1% formic acid, *v*/*v*) as solvent A. For analysis of compounds no. **1**–**14** (Figure 2), the following solvent gradient (0.5 mL/min) was applied: 0 min, 40% B; 0.1 min, 40% B; 1 min, 57.5% B; 3.5 min, 60% B; 4.25 min, 62.5% B; 6.25 min, 80% B; 6.5 min, 100% B; 7.2 min, 100% B; 7.25 min, 40% B; and 8 min, 40%. The mass spectrometer system was operated in low molecular mass configuration in the multiple reaction monitoring (MRM) and in the positive electrospray ionization (ESI^+^) mode using an ion spray voltage of 5.5 kV. The following source parameters were used given in parentheses: curtain gas (35 psi), nebulizer gas (55 psi), heater gas (65 psi), temperature 550 °C, collision activated dissociation (-3 V), and entrance potential (10 V). Quadrupoles operated at unit mass resolution. Compound optimization for declustering potential, collision energy, and cell exit potential as well as the MS/MS parameters of each substance (solved in 0.1% FA in acetonitrile/0.1% FA in water (70/30, *v*/*v*)) was carried out by direct flow infusion (10 µL/min). Optimization of ionization and fragmentation for sensitive MRM analysis was obtained by software-assisted ramping of the respective ion source and collision cell parameters. The mass transitions monitored and the respective ion source parameters are listed in Supplementary Table S3.

Statistical Analysis. Principal component analysis (PCA) was used for exploratory data analysis, allowing to visualize the variation present in a dataset using score plots labeled with field, genotype, biological replicates, and technical replicates. No major outliers were observed, and each data point was further used for data analysis. For overall analysis, we used pair wise Wilcoxon–Mann–Whitney test, using tidyverse package in R to find significantly different metabolites across different experimental conditions shown (Supplementary Table S4). However, the list of metabolites that are significantly different between genotypes are also provided as Supplementary Table S5. Data analysis was performed using R version 3.6.3 as well as Statistica 7.1 (www.statsoft.com (accessed on 7 January2021)). Fold change between two groups of log transformed data was computed by taking a ratio of median value across biological and technical replicates.

## 3. Results and Discussion

To characterize the influence of different water-stress conditions (WSC), namely waterlogging (wl) and drought (dr) (in comparison to the control (non-stressed, ns)), on the bitter sensometabolome of carrots, six distinct, orange carrot (*Daucus carota* spp. *sativus*) cultivars obtained from various collections (Figure 1 and Table 1) were grown and watered in a soil-seedbed under a plastic tunnel.

The ANOVA and Tukey analyses showed differences between the cultivars for the agronomical traits, and the different water supply (WSC) passed also to significant results (Supplementary Table S6). The increased water supply led in tendency to increased aerial and subaerial biomass in all cultivars. Under wl conditions, the cv Gosun (G5) showed an significant increased plant height (PH) and the cvs Texto (G1), Nagykallo (G2), Senta (G3), and Vita Longa (G6) an increased leaf biomass per plot (LM). For G3, also the calculated leaf mass per plant (ALM) was higher in comparison with the ns and dr variant. The number of harvested roots per plot (MR-n, Sum-m) were partially significant between the cultivars but not between the water applications for each cultivar. The mass of marketable roots (MR-m) and also the calculated (Sum-m) was in tendency the highest in the wl and lowest in the dr variant for cv Vita Longa (G6) significant.

In summary, the waterlogged plants frequently showed cracked and diseased carrots, but the additional water was not incorporated adequately in the root mass (Figure 2, left). Compared to the normally irrigated plant, drought-stressed carrots clearly revealed quality losses. The roots were partially smaller, reduced in weight, and the surface frequently puckered and constricted (Figure 2, right).

Recent investigations revealed compounds no. **1**–**14** (Figure 1) as the key compounds contributing to the bitter taste of carrots [3,4,5,6]. In order to investigate the biological fate of all these compounds in six different genotypes (Table 1) in response to different stress conditions, namely waterlogging (wl) and drought (dr), in comparison to the control (non-stressed, ns), reference compounds needed to be isolated. Closely following the procedure for the isolation reported in the literature, compounds **1**–**5** and **7**–**13** were isolated from carrots, respectively, and **6** as well as **14** were purchased [3,4,5,6].

Mass spectrometric method development was started by means of compound optimization of ionization and fragmentation for sensitive MRM analysis via software-assisted ramping of the respective ion source and collision cell parameters by direct flow infusion (Supplementary Table S3). Ethyl 3-(2H-1,3-benzodioxol-5-yl)-3-oxopropanoate (**IS-1**, for analytes **1**–**5**, **7**–**12**) and 5,7-dodecandiyn-1,2-diol (**IS-2**, for analytes **6**, **13**, **14**,) were commercially obtained and used as structurally related internal standards.

**Figure 3:**
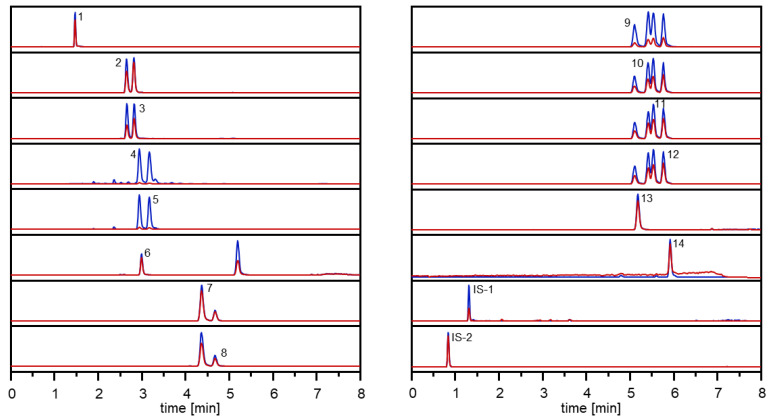
UHPLC-MS/MS analysis of compounds **1**–**14** in carrots.

Optimization of the chromatographic conditions enabled sufficient separation of **1**–**14** during UHPLC analysis in less than 8 min and their sensitive detection by means of MRM in the positive ESI mode (Figure 3).

In order to check the performance of the developed UHPLC-MS/MS method linearity, trueness, intraday and interday precision, sensitivity, and selectivity were investigated. To convert the measured mass transition ion intensities into the mass ratios of the internal standards and the analytes, graphs were calculated from calibration mixtures of known mass ratios and the corresponding peak area ratios in UHPLC-MS/MS (Supplementary Table S1). Calibration curves, obtained by linear regression analysis of the peak area vs. concentration, showed a linear response with a correlation coefficient of >0.98 (Supplementary Table S2). Carrots were extracted and spiked with a defined amount of the internal standards, followed by equilibration at room temperature. UHPLC-MS/MS analysis revealed strongly varying concentrations from 0.02–64.04 µg/g (Table 1). The lowest amount was detected for laserine oxide (**2**); most of the compounds highlighted concentrations in the range of 0.19–0.78 µg/g, followed by laserine (**7**) and 6,8-*O*-ditigloyl-6*β*,8α,11-trihydroxygermacra-1(10)*E*,4*E*-diene (**9**), with values above 1, 1.21, and 1.56 µg/g, respectively. Clearly higher amounts (11.25 and 12.43 µg/g) revealed the falcarinol-type polyacetylenes falcarinol (**14**), its diolacetate (**13**), and—by far the highest amount—falcarindiol (**6**), with 64.04 µg/g, which are in line with earlier investigations [4,18]. Busta et al. analysed tissue-specific accumulation of PAs in the orange cv Danvers and measured the highest total PA content in the peridermal tissues, with falcarindiol as the by far dominating PA [26]. In order to check the accuracy of the analytical method, recovery experiments were performed (Table 2). To achieve this, reference material of **1**–**14** was added to carrots in four different concentrations prior to quantitative analysis, and the amounts determined after clean up were compared with those found in the blank carrot sample. The recovery rates, calculated on the basis of the content of each bitter compound added to the carrots prior to clean up, were found to range between 80 and 120%, mainly 90–110% (Table 2).

Intraday as well as interday precision expressed as relative standard deviation (RSD) given in parentheses was quite similar, in a small range, and determined to be for compound **1** (2.3, 1.6%), **2** (4.4, 3.4%), **3** (3.1, 3.4%), **4** (2.1, 3.2%), **5** (2.0, 2.7%), **6** (1.6, 4.2%), **7** (13.5, 15.3%), **8** (2.9, 1.5%), **9** (3.0, 5.0%), **10** (3.7, 9.6%), **11** (3.8, 5.7%), **12** (5.0, 10.7%), **13** (2.0, 6.8%) and **14** (2.1, 5.4%). The lowest concentration of calibrated analytes **1**–**14** were all above the signal-to-noise ratio of 10 (Supplementary Table S2), and the selectivity of the method expressed as matrix interferences detected via the specific mass transitions of an analysed carrot sample was very good (Figure 3). These data clearly demonstrate the developed UHPLC-MS/MS method as a reliable tool enabling a rapid and accurate quantitative determination in carrots.

For overall analysis we have used pair wise Wilcoxon–Mann–Whitney test to find significantly different metabolites across different experimental conditions (Supplementary Table S4). Principal component analysis (PCA) was used for exploratory data analysis, allowing to visualize the variation present in a dataset with many variables, such as field, genotype, biological replicates, and technical replicates (Supplementary Figure S2a). No major outliers were observed, and each data point was further used for data analysis.

**Figure 4:**
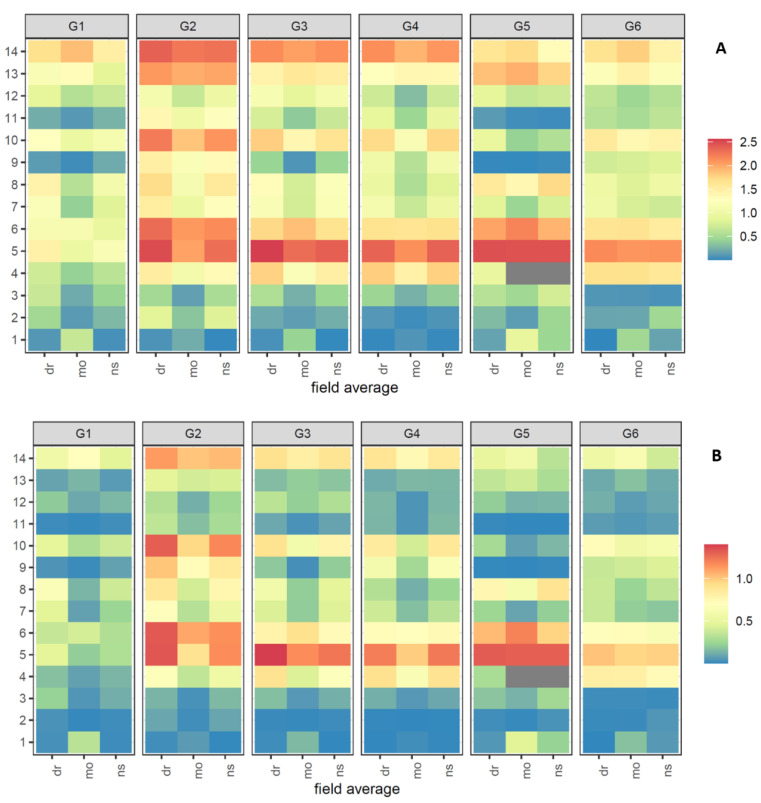
Heatmap presenting averaged (**A**) concentrations or (**B**) DoT-factors of metabolites **1**–**14** (as depicted in Figure 1) across three conditions of no stress (ns), waterlogging (wl), and drought stress (dr) over all fields (Figure 2).

The concentration of metabolites **1**–**14** across the three conditions of ns, wl, and dr and the six genotypes in fields a–c represented a very similar picture (Supplementary Figure S2a and S3) and are summarized as average in Figure 4. Surprisingly, **5** revealed by far the highest concentration, followed by the well-known bisacetylenes (**6**, **13**–**14**) but obviously not for all cultivars. The genotype G1 (Texto) indicated a very low concentration for **5** and, generally, lower concentrations for all metabolites compared to the other five cultivars. G2–G4 highlighted a similar pattern apart from single compounds like **13** and **10** in G2 (Nagykallo), and G5 (Vita Longa) displayed the highest amount for vaginatin (**5**) but the lowest for **9** and **11**.

In order to gain first insights into the bitter-taste impact of these compounds, these were rated on the basis of their dose-over-threshold (DoT) factors, defined as the ratio of the concentration and the taste-recognition threshold of a compound (Supplementary Table S7 and Figure S3 [27,28]. Calculation of the DoT factors revealed high values for **5** as well as bisacetylenes (**6**, **13**–**14**), but here, again, this is not consistent for all cultivars. In principle, caused by the low DoTs, the carrot “Texto” should be used for future cultivation and breeding studies when water stress is expected; in contrast, its opposite, G2, highlighted the highest DoT factors. The genotypes G3 and G4 also clearly displayed DoT above one, followed by G5 and G6. Future perspectives dealing with the bitter taste of carrots should include vaginatin (**5**) as an important contributor although strongly depending on the cultivar.

**Figure 5:**
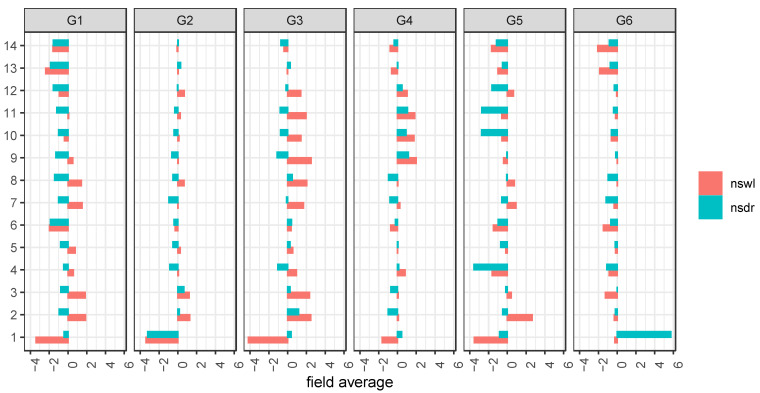
The bar plot presenting averaged (over all three fields) fold change (FC) between two comparisons, such as conditions no stress (ns) vs. waterlogging (wl) and drought stress (dr), respectively, for field a. The red bars represent ns vs. wl, and blues bar show FC between ns vs. dr, left side negatively/down- and right side positive/up-regulated.

In order to bridge the gap between the concentrations of the metabolites **1**–**14** and their fates during the different stress conditions, fold changes (FC) comparing the two stress conditions, namely drought (dr) and waterlogging (wl), vs. control (ns) were calculated for each individual field (Supplementary Figure S4) as well as an average (Figure 5). 6-methoxymellein (**1**) revealed the highest FC for all metabolites, and its fate is not clearly dependent on the stress condition. Recently, higher levels of **1** were correlated with a higher level of spontaneous infection of carrot leaves (*Alternaria dauci*, *Cercospora carotae,* and the bacterium *Xanthomonas campestris pv. carotae* but were not distinguished between the effects of the individual pathogens) that were not treated with fungicide, but our data showed that **1** was down-regulated under waterlogging in all genotypes [29]. Waterlogging could be expected with a higher infection, but further influences may have stronger impact, e.g., the FC during drought vs. non-stressed for **1** indicated three times up- and down-regulation, respectively. Additionally, the impact of the FC is pronounced differently in all cultivars. A closer look at the prominent bisacetylenes (**6**, **13**–**14**) elucidates that the compound class behaves in all genotypes very similarly, and mostly were down-regulated for both stress conditions, but again, the impact is strongly different. Additionally, the cultivars G2 and G3 indicated minor up-regulation. Further, the compound classes of the laserin derivatives (**2**, **3**, **7**, **8**) as well as trihydroxygermacradienes (**9**–**12**) highlighted a mixed behavior under drought and waterlogging compared to non-stressed.

## 4. Conclusions

In summary, a fast and robust high-throughput UHPLC-MS/MS was developed and validated enabling tracing the metabolic changes of carrots bitter compounds. The method was successfully applied to six carrot cultivars in from our randomized block field trial with three irrigation conditions (waterlogging, drought, and control) and three biological replicates. After sample preparation, the sensometabolites were quantified in triplicate. Statistical analysis of the concentrations obtained revealed no major outliers, and each data point was further used for data analysis. Surprisingly, the genotypes are the driving source for the biological fate of the bitter metabolites that are reflected in concentrations, DoT-factors, and FC. A certain influence of the irrigation level is observable but overruled by its cultivar. In sum, metabolic stress response in carrots seems to be genotype dependent. The collected knowledge of this study might help to breed and plant specific carrot genotypes that might efficiently enhance or sustain the commercial production under future climatic stress conditions.

## Figures and Tables

**Table 1:** Plant material used in this study.

Genotype	Cultivar	Source ^1^	Origin	Breeding Status ^2^
G1	Texto	IRHS	FRA	F_1_
G2	Nagykallo	WGRU 5779	HUN	OP
G3	Senta	DEU 437	DEU	OP
G4	Delta a Cuore Rosso	WGRU 7125	ITA	OP
G5	Himuro Fuyugosi Gosun No.2	WGRU 11718	JPN	OP
G6	Vita Longa	Bejo	NLD	OP

^1^ IRHS—Agrocampus Ouest, Angers, (FRA); Bejo—Bejo Zaden B.V., Warmenhuizen, (NLD); DEU—The Leibniz Institute of Plant Genetics and Crop Plant Research (IPK), Gatersleben (DEU); WGRU—Warwick Genetic Resources Unit, Warwick University, Wellesbourne (UK);.^2^ OP—Open pollinated cultivar, F_1_—hybrid cultivar.

**Table 2:** Determination of the recovery rates for the quantitative analysis of bitter compounds **1**–**14** in carrots.

No. of	Amount Added	Conc. Calculated	Conc. Determined	Recovery [%]
Cmpd.	[µg/g]	[µg/g]	[µg/g]	
**1**	-	-	0.19	-
	0.04	0.23	0.21	90.0
	0.08	0.27	0.28	104.5
	0.17	0.36	0.24	67.2
	0.42	0.61	0.35	56.9
**2**	-	-	0.02	-
	0.08	0.10	0.10	96.0
	0.16	0.18	0.15	84.5
	0.32	0.34	0.34	99.3
	0.81	0.83	0.87	104.8
**3**	-	-	0.27	
	0.08	0.35	0.42	120.6
	0.17	0.43	0.40	93.2
	0.33	0.60	0.57	94.7
	0.83	1.09	0.95	86.9
**4**	-	-	0.21	-
	0.07	0.28	0.26	93.9
	0.13	0.35	0.29	83.3
	0.27	0.48	0.38	79.5
	0.67	0.88	0.68	76.8
**5**	-	-	0.78	-
	0.07	0.85	0.98	115.9
	0.13	0.91	0.90	98.3
	0.26	1.05	0.85	80.9
	0.66	1.44	1.09	75.6
**6**	-	-	64.04	-
	0.05	64.09	64.79	101.1
	0.10	64.14	73.91	115.2
	0.20	64.24	70.19	109.3
	0.51	64.55	62.44	96.7
**7**	-	-	1.21	-
	0.08	1.29	1.45	112.4
	0.16	1.37	1.30	94.7
	0.32	1.53	1.99	130.0
	0.80	2.01	2.49	124.1
**8**	-	-	0.35	-
	0.08	0.43	0.45	105.4
	0.16	0.51	0.55	107.9
	0.31	0.67	0.64	96.8
	0.78	1.13	0.96	84.2
**9**	-	-	1.56	-
	0.08	1.64	1.72	104.6
	0.17	1.73	1.93	112.0
	0.33	1.89	2.25	118.7
	0.83	2.39	1.50	63.0
**10**	-	-	0.18	-
	0.04	0.23	0.23	103.3
	0.08	0.27	0.29	107.1
	0.17	0.35	0.34	95.5
	0.42	0.60	0.49	81.0
**11**	-	-	0.18	-
	0.08	0.26	0.26	99.7
	0.17	0.35	0.36	103.5
	0.34	0.52	0.43	82.9
	0.84	1.02	0.67	65.8
**12**	-	-	0.25	-
	0.08	0.33	0.30	91.5
	0.17	0.41	0.39	93.3
	0.33	0.58	0.49	85.2
	0.84	1.08	0.77	70.9
**13**	-	-	12.43	-
	0.06	12.30	14.19	115.4
	0.12	12.36	14.09	114.0
	0.24	12.48	15.80	126.6
	0.60	12.84	15.77	122.8
**14**	-	-	11.25	-
	0.05	11.29	14.72	130.4
	0.09	11.34	10.42	91.9
	0.19	11.43	8.21	71.8
	0.47	11.71	13.82	118.0

## Data Availability

Data is contained within the article as well as supplementary material.

## Supplementary Materials

The following are available online at https://www.mdpi.com/article/10.3390/foods10071607/s1, Figure S1: The six carrots cultivars growing in the soil-seedbed under a plastic tunnel (Rain-out–shelter) drop irrigated by using a sprinkler hose system. Figure S2a. Score plots of principal component analysis (PCA) of metabolite data. PCA score plot of PC1 versus PC2 discriminating the genotypes. Figure S2b. Heatmap presenting concentration of metabolites 1–14 (as depicted in Figure 1) across three conditions no stress (ns), waterlogging (wl) and drought stress (dr) from fields a-c. Figure S3. Heatmap presenting concentration of metabolites 1–14 (as depicted in Figure 1) across three conditions no stress (ns), waterlogging (wl) and drought stress (dr) from fields a-c. The star presents the DoT information (one star is DoT > 0.1, two stars is 0.1 < DoT < 10 and three stars are DoT values > 10). Figure S4. The bar plot presenting fold change (FC) between two comparisons such as conditions no stress (ns) vs waterlogging (wl) and drought stress (dr) respectively for field a, b and c. The red bar shows fold change between ns vs wl and blue bar shows FC between ns vs dr. The bar on left are the negatively / down regulated whereas the bar on right shows the positive/ up regulation. Table S1: Concentrations (µmol/L) of calibration solutions of compounds 1–14. Table S2: Calibration functions of single analytes and correlation coefficients as well signal-to-noise value of lowest calibration concentration. Table S3: MRM transitions and optimized MS/MS parameters of analyzed compounds 1–14. Table S4: Quantification of compounds 1–14 across 6 genotypes, 3 conditions (ns, wl, dr) and field a, b and c. The star presents the DoT information (one star is DoT > 0.1, two stars is 0.1 < DoT < 10, three stars are DoT values > 10 and NA to DoT value < 0.1). Table S5: Overall analysis using pair wise Wilcoxon-Mann-Whitney-Test to find significantly different metabolites across different experimental conditions. Table S6: The ANOVA and Tukey analyses showed differences between the cultivars (CV) and the different water supply (WSC) for the expression of the agronomical traits (bold letters). Table S7: Taste thresholds of compounds 1–14.
