# Supplementary material for: Influence of the Abiotic Stress Conditions, Waterlogging and Drought, on the Bitter Sensometabolome as Well as Agronomical Traits of Six Genotypes of Daucus carota
Source: Foods. 2021 Jul 11;10(7):1607. doi: 10.3390/foods10071607 (PMC8305268; doi:10.3390/foods10071607)
Supplement: Supplementary file 1 [file foods-10-01607-s001.zip › foods-1265614-supplementary materials.pdf]

Supplementary Materials

Influence of the Abiotic Stress Conditions, Waterlogging and Drought, on the Bitter Sensometabolome as Well as Agronomical Traits of Six Genotypes of *Daucus carota*

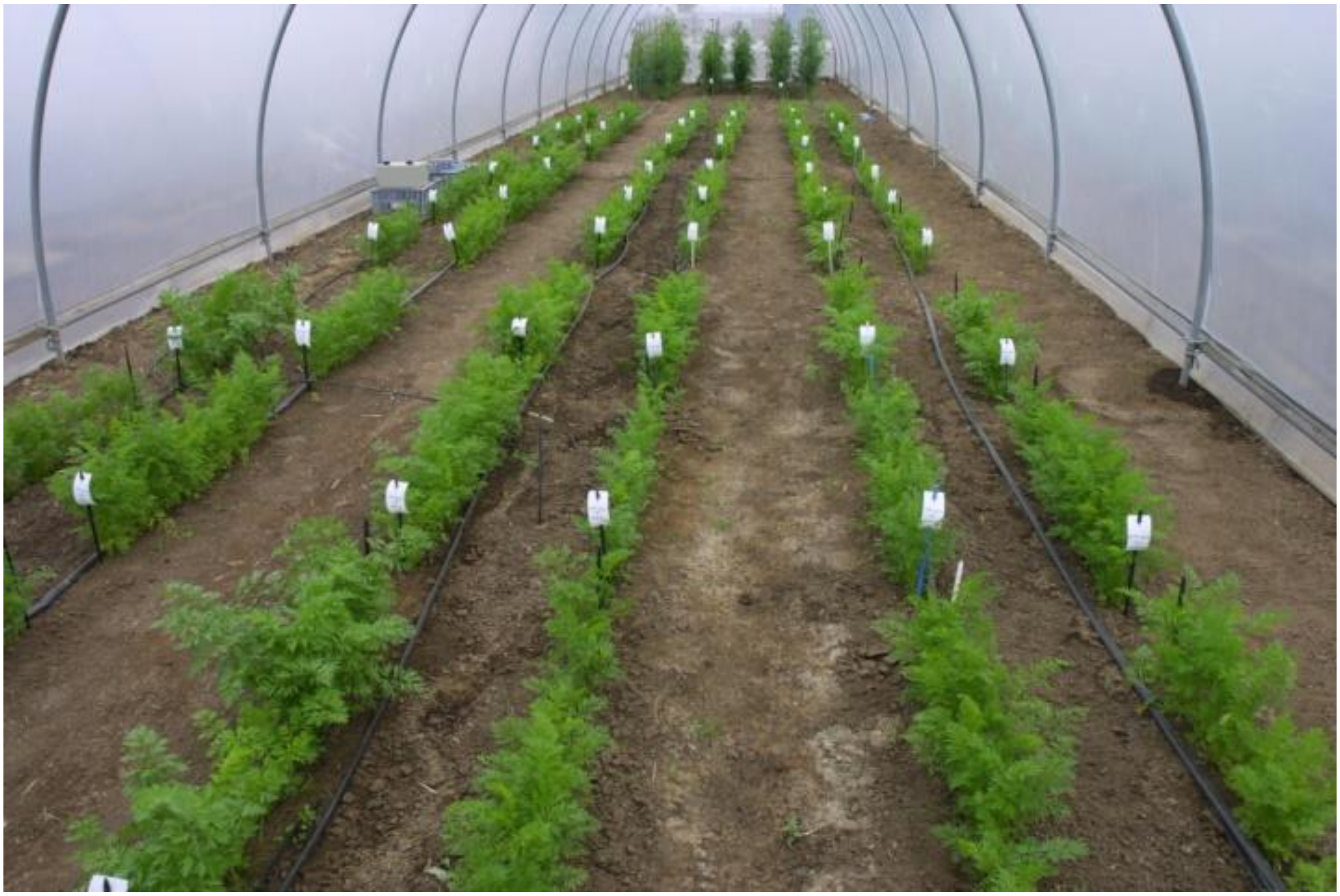

Figure S1. The six carrots cultivars growing in the soil-seedbed under a plastic tunnel (Rain-out–shelter) drop irrigated by using a sprinkler hose system.

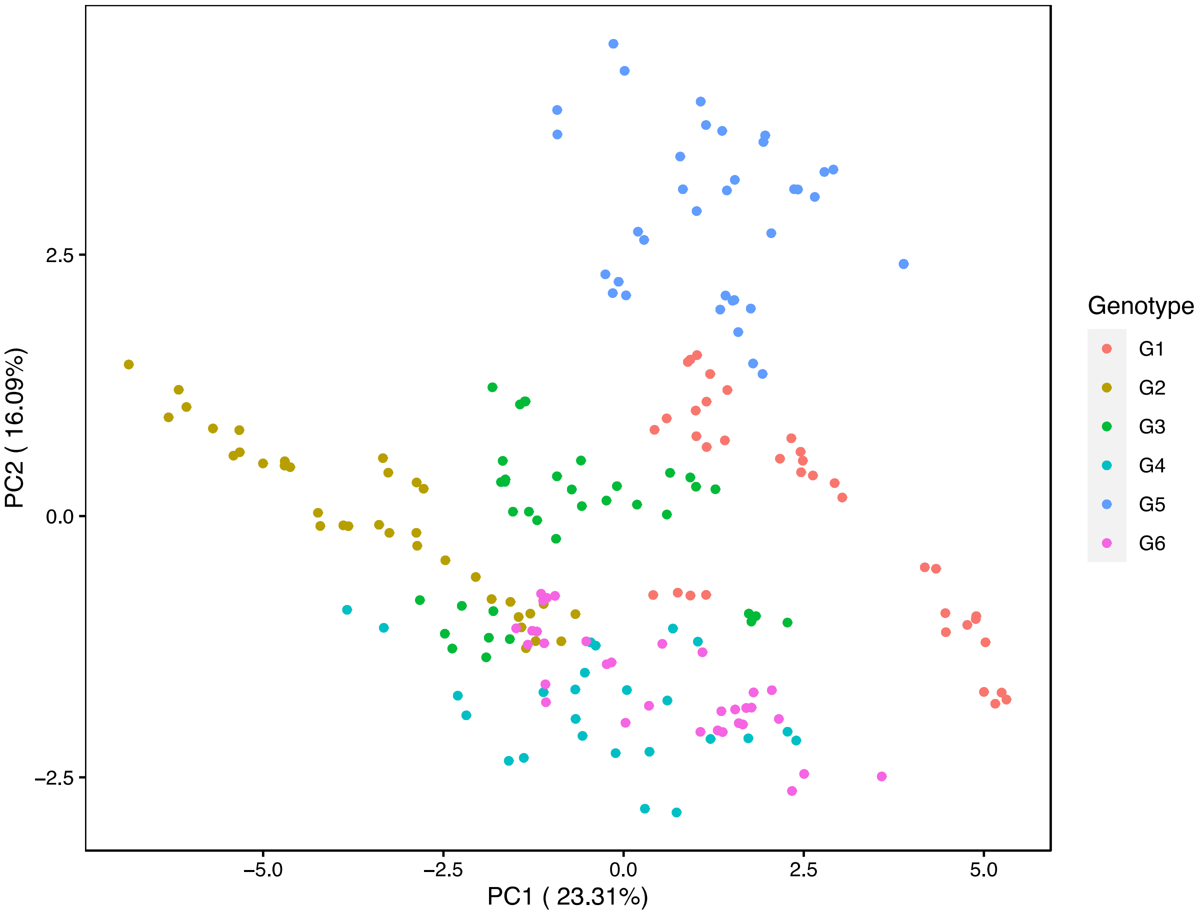

Figure S2a. Score plots of principal component analysis (PCA) of metabolite data. PCA score plot of PC1 versus PC2 discriminating the genotypes.

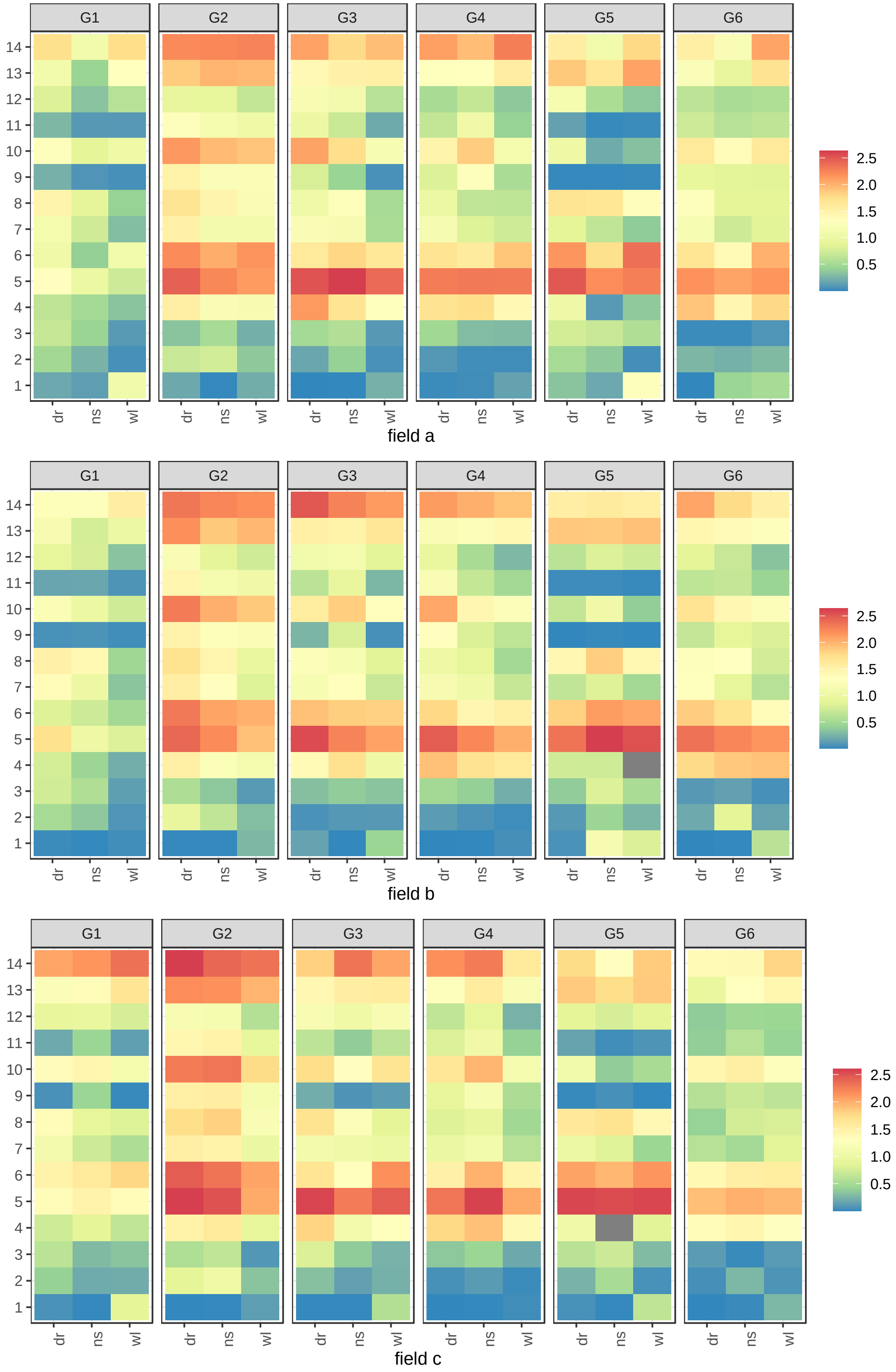

Figure S2b. Heatmap presenting concentration of metabolites **1-14** (as depicted in Figure 1) across three conditions no stress (ns), waterlogging (wl) and drought stress (dr) from fields a-c.

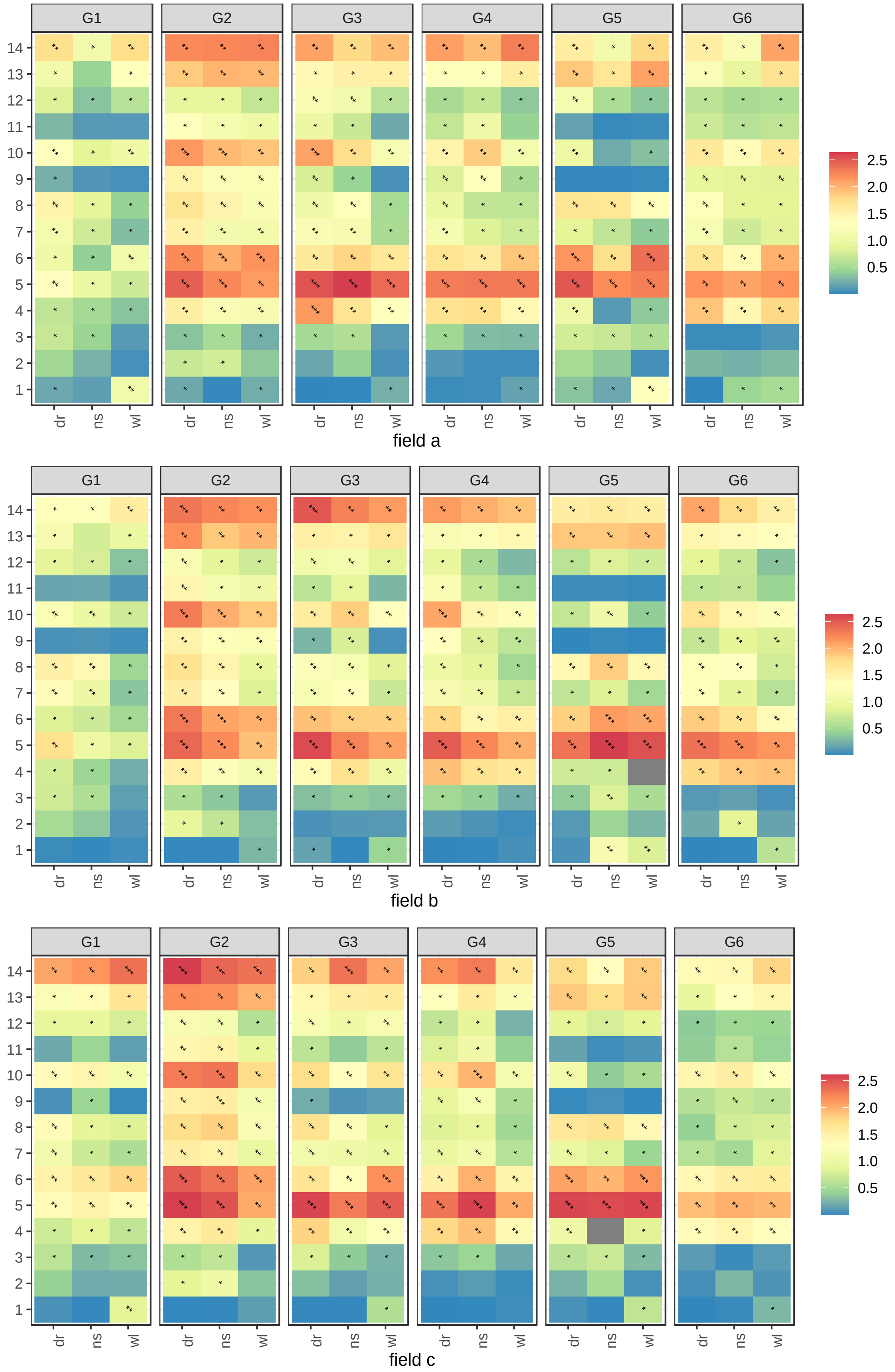

Figure S3. Heatmap presenting concentration of metabolites **1-14** (as depicted in Figure 1) across three conditions no stress (ns), waterlogging (wl) and drought stress (dr) from fields a-c. The star presents the DoT information (one star is Dot >0.1, two stars is Dot >0.1 <10 and three stars are Dot values >10).

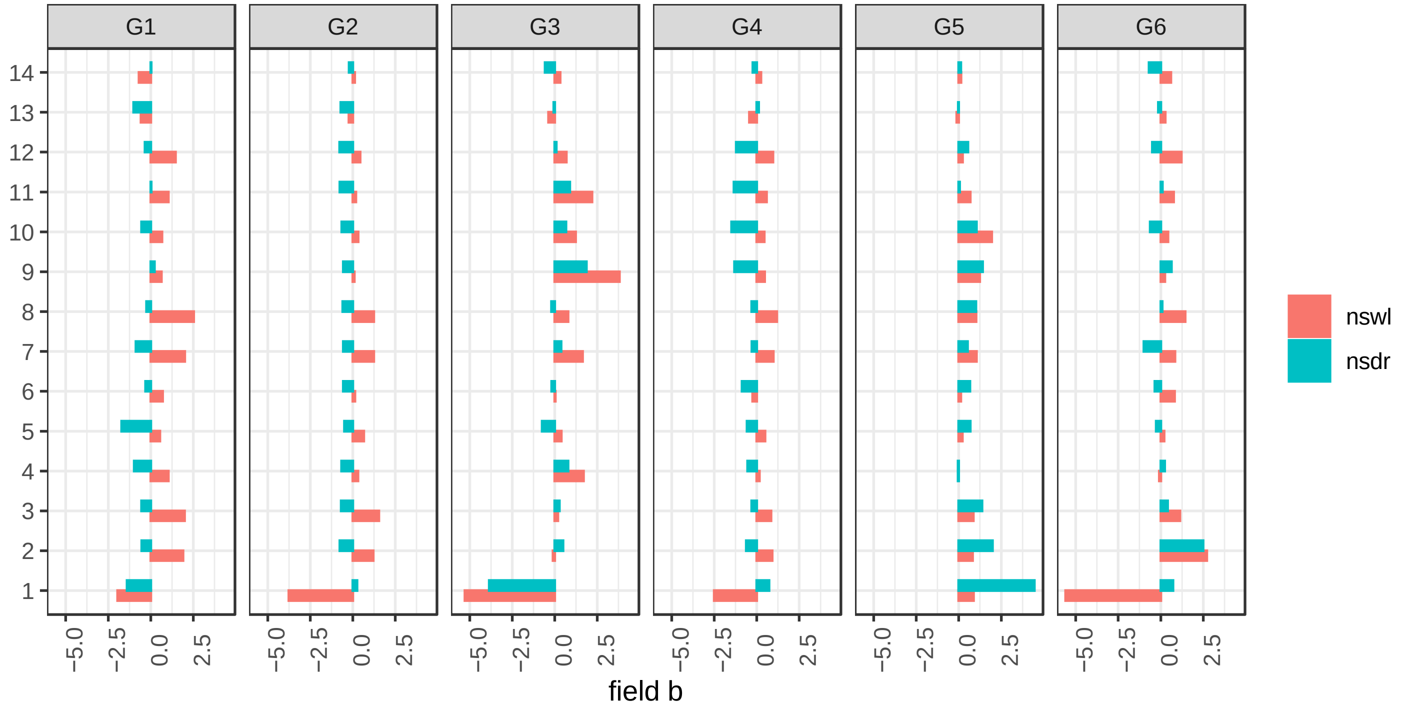
G1
G2
G3
G4
G5
G6
14
13
12
11
10
9
8
7
6
5
4
3
2
1
−5.0
−2.5
0.0
2.5
field b
nswl
nsdr

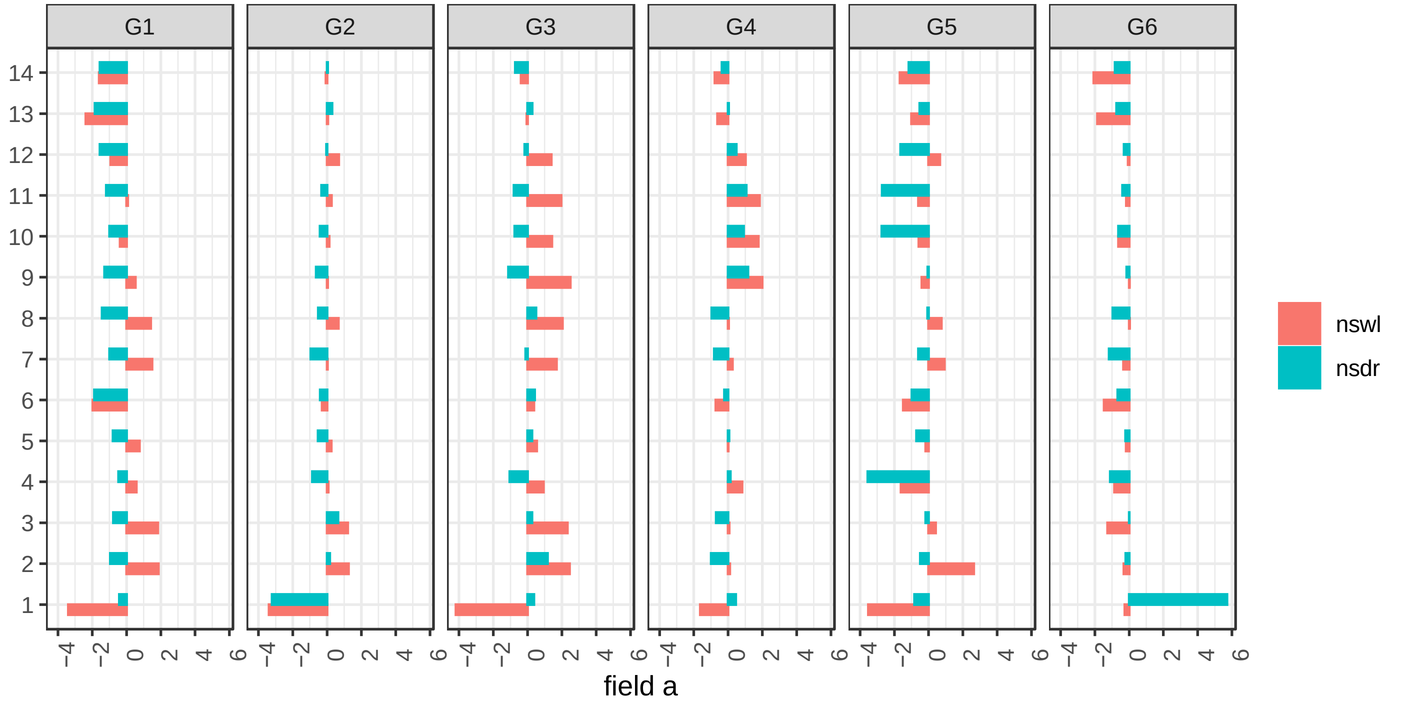
G1
G2
G3
G4
G5
G6
14
13
12
11
10
9
8
7
6
5
4
3
2
1
−4
−2
0
2
4
6
field a
nswl
nsdr

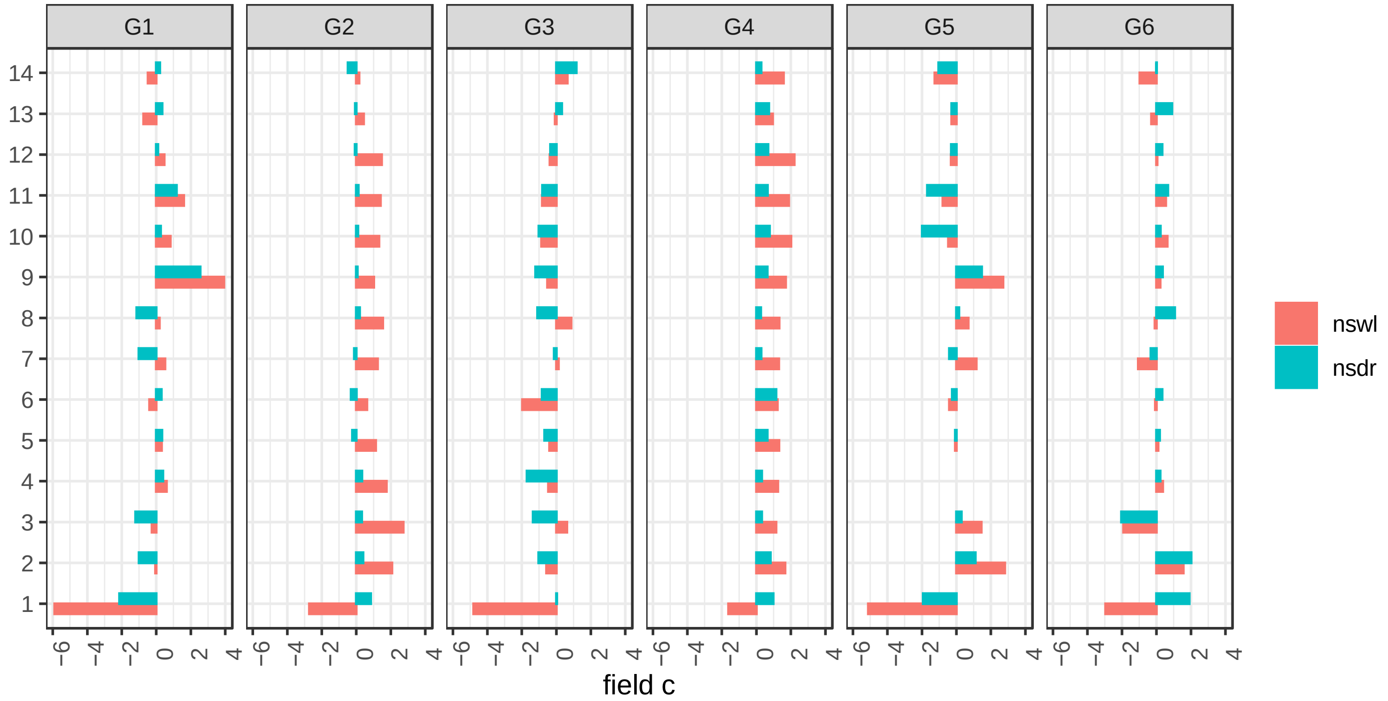

Figure S4. The bar plot presenting fold change (FC) between two comparisons such as conditions no stress (ns) vs waterlogging (wl) and drought stress (dr) respectively for field a, b and c. The red bar shows fold change between ns vs wl and blue bar shows FC between ns vs dr. The bar on left are the negatively / down regulated whereas the bar on right shows the positive/ up regulation.

Table S1: Concentrations (μmol/L) of calibration solutions of compounds 1-14.

Compound No.													
**1**	**2**	**3**	**4**	**5**	**6**	**7**	**8**	**9**	**10**	**11**	**12**	**13**	**14**
20.378	19.976	20.316	19.968	19.600	19.656	20.549	20.028	19.712	9.984	20.056	19.980	20.000	10.032
10.189	9.988	10.158	9.984	9.800	9.828	10.274	10.014	9.856	4.992	10.028	9.990	10.000	5.016
5.095	4.994	5.079	4.992	4.900	4.914	5.137	5.007	4.928	2.496	5.014	4.995	5.000	2.508
2.038	1.998	2.032	1.997	1.960	1.966	2.055	2.003	1.971	0.998	2.006	1.998	2.000	1.003
1.019	0.999	1.016	0.998	0.980	0.983	1.027	1.001	0.986	0.499	1.003	0.999	1.000	0.502
0.509	0.499	0.508	0.499	0.490	0.491	0.514	0.501	0.493	0.250	0.501	0.500	0.500	0.251
0.204	0.200	0.203	0.200	0.196	0.197	0.205	0.200	0.197	0.100	0.201	0.200	0.200	0.100
0.102	0.100	0.102	0.100	0.098	0.098	0.103	0.100	0.099	0.050	0.100	0.100	0.100	0.050
0.051	0.050	0.051	0.050	0.049	0.049	0.051	0.050	0.049	0.025	0.050	0.050	0.050	
0.020	0.020	0.020	0.020	0.020	0.020	0.021	0.020	0.020	0.010	0.020	0.020	0.020	
0.010	0.010	0.010	0.010	0.010		0.010	0.010	0.010	0.005	0.010	0.010		
0.005	0.005	0.005	0.005	0.005		0.005	0.005	0.005	0.002	0.005	0.005		
0.002	0.002	0.002	0.002	0.002		0.002	0.002	0.002	0.001	0.002	0.002		
0.001	0.001	0.001	0.001	0.001		0.001	0.001		0.000	0.001	0.001		

Table S2: Calibration functions of single analytes and correlation coefficients as well signal-to-noise value of lowest calibration concentration.

	**Calibration Function**	**r2**	**Signal to Noise Lowest Conc**
**1**	y=0.35176x-0.00116	0.98	21.0
**2**	y=0.46097x-0.00219	0.98	46.9
**3**	y=0.13283x-0.000403	0.99	30.4
**4**	y=0.12563x-0.0002394	0.99	23.1
**5**	y=0.99353x-0.04791	0.99	91.9
**6**	y=0.03191x+0.00207	0.99	52.9
**7**	y=0.26467x-0.00124	0.98	25.1
**8**	y=0.10374x-0.00876	0.99	14.5
**9**	y=0.06595x-0.00445	0.99	10.0
**10**	y=1.0307x-0.00191	0.98	80.2
**11**	y=0.79142x-0.00266	0.99	109.3
**12**	y=0.60861x-0.0023	0.99	102.6
**13**	y=0.07317x+0.00303	0.99	122.0
**14**	y=0.06204x+0.0002484	0.99	13.5

Table S3: MRM transitions and optimized MS/MS parameters of analyzed compounds **1-14**.

Compound (no.)	Q1	Q3	DP	CE	CXP
6-methoxymellein (**1**) quant	209.1	191	86	21	22
6-methoxymellein (**1**) qual	209.1	163	86	27	18
laserine oxide (**2**) quant	429.1	328.9	106	21	40
laserine oxide (**2**) qual	429.1	291	106	19	34
2-epilaserine oxide (**3**) quant	429.1	328.9	176	21	40
2-epilaserine oxide (**3**) qual	429.1	291	176	19	34
isovaginatin (**4**) quant	357.1	257.1	186	19	24
isovaginatin (**4**) qual	357.1	119	186	47	14
vaginatin (**5**) quant	357.1	257.1	66	19	30
vaginatin (**5**) qual	357.1	90.9	66	77	10
falcarindiol (**6**) quant	243	91	21	31	8
falcarindiol (**6**) qual	243	77.1	21	57	10
laserine (**7**) quant	413	291	71	17	34
laserine (**7**) qual	413	83	71	21	10
2-epilaserine (**8**) quant	413	291	71	17	34
2-epilaserine (**8**) qual	413	83	71	21	10
6,8-*O*-ditigloyl-6β,8α,11-trihydroxygermacra-1(10)E,4E-diene (**9**) quant	441.2	283.1	186	19	34
6,8-*O*-ditigloyl-6β,8α,11-trihydroxygermacra-1(10)E,4E-diene (**9**) qual	441.2	341.1	186	19	12
6-O-angeloyl-8-O-tigloyl-6β,8α,11-trihydroxygermacra-1(10)E,4E-diene (**10**) quant	441.2	283.1	71	19	32
6-O-angeloyl-8-O-tigloyl-6β,8α,11-trihydroxygermacra-1(10)E,4E-diene (**10**) qual	441.2	341.2	71	19	38
6-*O*-tigloyl-8-*O*-angeloyl-6β,8α,11-trihydroxygermacra-1(10)E,4E-diene (**11**) quant	441.2	283.1	81	17	32
6-*O*-tigloyl-8-*O*-angeloyl-6β,8α,11-trihydroxygermacra-1(10)E,4E-diene (**11**) qual	441.2	341	81	19	12
6,8-*O*-diangeloyl-6β,8α,11-trihydroxygermacra-1(10)E,4E-diene (**12**) quant	441.2	283	76	19	34
6,8-*O*-diangeloyl-6β,8α,11-trihydroxygermacra-1(10)E,4E-diene (**12**) qual	441.2	341.2	76	19	12
falcarindiol-3-acetate (**13**) quant	285	144.9	51	23	18
falcarindiol-3-acetate (**13**) qual	285	115	51	49	14
falcarinol (**14**) quant	227	129	41	27	14

falcarinol (**14**) qual	227	90.9	41	33	10
ethyl 3-(2H-1,3-benzodioxol-5-yl)-3-oxopropanoate (**IS-1**) quant	237	148.9	51	19	16
ethyl 3-(2H-1,3-benzodioxol-5-yl)-3-oxopropanoate (**IS-1**) qual	237	120.9	51	45	14
5,7-Dodecandiyn-1,2-diol (**IS-2**) quant	195.1	125	51	21	14
5,7-Dodecandiyn-1,2-diol (**IS-2**) qual	195.1	69	51	23	8

Table S4: Quantification of compounds **1-14** across 6 genotypes, 3 conditions (ns, wl, dr) and field a, b and c. The star presents the DoT information (one star is Dot >0.1, two stars is Dot >0.1 <10, three stars are Dot values >10 and NA to Dot value < 0.1).

Genotype	Condition	Field	Compounds	Median_value	Median_Dotvalue	Median_logvalue	Median_logDotvalue	Stars
G1	ns	a	3	1.79	0.32	0.45	0.12	*
G1	wl	a	3	0.29	0.05	0.11	0.02	NA
G1	dr	a	3	3.87	0.68	0.69	0.23	*
G2	ns	a	3	2.35	0.41	0.52	0.15	*
G2	wl	a	3	0.7	0.12	0.23	0.05	*
G2	dr	a	3	1.24	0.22	0.35	0.09	*
G3	ns	a	3	2.82	0.49	0.58	0.17	*
G3	wl	a	3	0.28	0.05	0.11	0.02	NA
G3	dr	a	3	2.19	0.38	0.5	0.14	*
G4	ns	a	3	1.01	0.18	0.3	0.07	*
G4	wl	a	3	0.95	0.17	0.29	0.07	*
G4	dr	a	3	2.02	0.36	0.48	0.13	*
G5	ns	a	3	4.1	0.72	0.71	0.24	*
G5	wl	a	3	2.72	0.48	0.57	0.17	*
G5	dr	a	3	4.89	0.86	0.77	0.27	*
G6	ns	a	3	0.06	0.01	0.02	0	NA
G6	wl	a	3	0.2	0.04	0.08	0.01	NA
G6	dr	a	3	0.06	0.01	0.02	0	NA
G1	ns	a	1	0.37	0.09	0.14	0.04	NA
G1	wl	a	1	11.01	2.64	1.08	0.56	**
G1	dr	a	1	0.56	0.13	0.19	0.05	*
G2	ns	a	1	0.02	0.01	0.01	0	NA
G2	wl	a	1	0.67	0.16	0.22	0.06	*
G2	dr	a	1	0.56	0.14	0.19	0.06	*
G3	ns	a	1	0.01	0	0	0	NA
G3	wl	a	1	0.74	0.18	0.24	0.07	*
G3	dr	a	1	0.01	0	0	0	NA
G4	ns	a	1	0.08	0.02	0.03	0.01	NA

G4	wl	a	1	0.43	0.1	0.15	0.04	*
G4	dr	a	1	0.05	0.01	0.02	0.01	NA
G5	ns	a	1	0.54	0.13	0.19	0.05	*
G5	wl	a	1	18.23	4.38	1.28	0.73	**
G5	dr	a	1	1.24	0.3	0.35	0.11	*
G6	ns	a	1	1.83	0.44	0.45	0.16	*
G6	wl	a	1	2.35	0.56	0.53	0.19	*
G6	dr	a	1	0.01	0	0	0	NA
G1	ns	a	11	0.28	0.01	0.11	0.01	NA
G1	wl	a	11	0.26	0.01	0.1	0.01	NA
G1	dr	a	11	0.91	0.05	0.28	0.02	NA
G2	ns	a	11	12.94	0.66	1.14	0.22	*
G2	wl	a	11	9.97	0.51	1.04	0.18	*
G2	dr	a	11	17.98	0.91	1.28	0.28	*
G3	ns	a	11	4.16	0.21	0.71	0.08	*
G3	wl	a	11	0.59	0.03	0.2	0.01	NA
G3	dr	a	11	9.25	0.47	1.01	0.17	*
G4	ns	a	11	10.63	0.54	1.07	0.19	*
G4	wl	a	11	1.7	0.09	0.43	0.04	NA
G4	dr	a	11	3.68	0.19	0.67	0.07	*
G5	ns	a	11	0.03	0	0.01	0	NA
G5	wl	a	11	0.05	0	0.02	0	NA
G5	dr	a	11	0.42	0.02	0.15	0.01	NA
G6	ns	a	11	2.97	0.15	0.6	0.06	*
G6	wl	a	11	3.49	0.18	0.65	0.07	*
G6	dr	a	11	4.36	0.22	0.73	0.09	*
G1	ns	a	14	90.15	25.57	1.96	1.42	***
G1	wl	a	14	428.73	121.62	2.63	2.09	***
G1	dr	a	14	767.55	217.73	2.89	2.34	***
G2	ns	a	14	541.26	153.54	2.73	2.19	***
G2	wl	a	14	197.98	56.16	2.3	1.76	***
G2	dr	a	14	893.04	253.33	2.95	2.41	***

G3	ns	a	14	3277.54	929.75	3.52	2.97	***
G3	wl	a	14	1325.78	376.09	3.12	2.58	***
G3	dr	a	14	4868.64	1381.11	3.69	3.14	***
G4	ns	a	14	122.76	34.82	2.09	1.55	***
G4	wl	a	14	77.12	21.88	1.89	1.36	***
G4	dr	a	14	101.18	28.7	2.01	1.47	***
G5	ns	a	14	122.82	34.84	2.09	1.55	***
G5	wl	a	14	54.25	15.39	1.74	1.21	***
G5	dr	a	14	554.47	157.29	2.74	2.2	***
G6	ns	a	14	102.83	29.17	2.02	1.48	***
G6	wl	a	14	520.01	147.51	2.72	2.17	***
G6	dr	a	14	417.7	118.49	2.62	2.08	***
G1	ns	a	12	1.2	0.11	0.34	0.04	*
G1	wl	a	12	3.03	0.27	0.61	0.1	*
G1	dr	a	12	5.64	0.5	0.82	0.18	*
G2	ns	a	12	7.33	0.65	0.92	0.22	*
G2	wl	a	12	3.72	0.33	0.67	0.12	*
G2	dr	a	12	7.55	0.67	0.93	0.22	*
G3	ns	a	12	12.11	1.07	1.12	0.32	**
G3	wl	a	12	3.04	0.27	0.61	0.1	*
G3	dr	a	12	14.4	1.27	1.19	0.36	**
G4	ns	a	12	3.83	0.34	0.68	0.13	*
G4	wl	a	12	1.38	0.12	0.38	0.05	*
G4	dr	a	12	2.39	0.21	0.53	0.08	*
G5	ns	a	12	2.52	0.22	0.55	0.09	*
G5	wl	a	12	1.31	0.12	0.36	0.05	*
G5	dr	a	12	12.94	1.14	1.14	0.33	**
G6	ns	a	12	2.49	0.22	0.54	0.09	*
G6	wl	a	12	2.64	0.23	0.56	0.09	*
G6	dr	a	12	3.35	0.3	0.64	0.11	*
G1	ns	a	9	0.2	0.06	0.08	0.03	NA
G1	wl	a	9	0.12	0.04	0.05	0.02	NA

G1	dr	a	9	0.73	0.22	0.24	0.09	*
G2	ns	a	9	15.95	4.76	1.23	0.76	**
G2	wl	a	9	15.33	4.58	1.21	0.75	**
G2	dr	a	9	30.11	8.99	1.49	1	**
G3	ns	a	9	1.75	0.52	0.44	0.18	*
G3	wl	a	9	0.14	0.04	0.06	0.02	NA
G3	dr	a	9	5.35	1.6	0.8	0.41	**
G4	ns	a	9	17.99	5.37	1.28	0.8	**
G4	wl	a	9	2.48	0.74	0.54	0.24	*
G4	dr	a	9	5.66	1.69	0.82	0.43	**
G5	ns	a	9	0.02	0.01	0.01	0	NA
G5	wl	a	9	0.03	0.01	0.01	0	NA
G5	dr	a	9	0.02	0.01	0.01	0	NA
G6	ns	a	9	6.42	1.92	0.87	0.47	**
G6	wl	a	9	6.3	1.88	0.86	0.46	**
G6	dr	a	9	7.37	2.2	0.92	0.51	**
G1	ns	a	6	1.59	0.15	0.41	0.06	*
G1	wl	a	6	11.33	1.09	1.09	0.32	**
G1	dr	a	6	10.41	1	1.06	0.3	*
G2	ns	a	6	107.44	10.32	2.04	1.05	***
G2	wl	a	6	143.73	13.8	2.16	1.17	***
G2	dr	a	6	160.07	15.37	2.21	1.21	***
G3	ns	a	6	62.96	6.05	1.81	0.85	**
G3	wl	a	6	43.72	4.2	1.65	0.72	**
G3	dr	a	6	41.73	4.01	1.63	0.7	**
G4	ns	a	6	38.36	3.68	1.6	0.67	**
G4	wl	a	6	78.44	7.53	1.9	0.93	**
G4	dr	a	6	47.64	4.57	1.69	0.75	**
G5	ns	a	6	53.86	5.17	1.74	0.79	**
G5	wl	a	6	236.12	22.67	2.37	1.37	***
G5	dr	a	6	142.32	13.66	2.16	1.17	***
G6	ns	a	6	23.76	2.28	1.39	0.52	**

G6	wl	a	6	102.12	9.8	2.01	1.03	**
G6	dr	a	6	46.28	4.44	1.67	0.74	**
G1	ns	a	4	2.19	0.25	0.5	0.1	*
G1	wl	a	4	1.23	0.14	0.35	0.06	*
G1	dr	a	4	3.49	0.4	0.65	0.15	*
G2	ns	a	4	14.52	1.67	1.19	0.43	**
G2	wl	a	4	13.58	1.56	1.16	0.41	**
G2	dr	a	4	34.46	3.96	1.55	0.7	**
G3	ns	a	4	47.88	5.51	1.69	0.81	**
G3	wl	a	4	18.98	2.18	1.3	0.5	**
G3	dr	a	4	135.2	15.55	2.13	1.22	***
G4	ns	a	4	56.25	6.47	1.76	0.87	**
G4	wl	a	4	24.86	2.86	1.41	0.59	**
G4	dr	a	4	49.27	5.67	1.7	0.82	**
G5	ns	a	4	0.28	0.03	0.11	0.01	NA
G5	wl	a	4	1.41	0.16	0.38	0.07	*
G5	dr	a	4	9.78	1.12	1.03	0.33	**
G6	ns	a	4	26.2	3.01	1.43	0.6	**
G6	wl	a	4	61.42	7.06	1.8	0.91	**
G6	dr	a	4	79.15	9.1	1.9	1	**
G1	ns	a	7	4.56	0.57	0.75	0.2	*
G1	wl	a	7	1.03	0.13	0.31	0.05	*
G1	dr	a	7	12.24	1.54	1.12	0.4	**
G2	ns	a	7	12.12	1.52	1.12	0.4	**
G2	wl	a	7	11.86	1.49	1.11	0.4	**
G2	dr	a	7	31.49	3.95	1.51	0.69	**
G3	ns	a	7	13.43	1.69	1.16	0.43	**
G3	wl	a	7	2.47	0.31	0.54	0.12	*
G3	dr	a	7	15.08	1.89	1.21	0.46	**
G4	ns	a	7	5.81	0.73	0.83	0.24	*
G4	wl	a	7	4.53	0.57	0.74	0.2	*
G4	dr	a	7	13.03	1.64	1.15	0.42	**

G5	ns	a	7	3.59	0.45	0.66	0.16	*
G5	wl	a	7	1.43	0.18	0.39	0.07	*
G5	dr	a	7	6.55	0.82	0.88	0.26	*
G6	ns	a	7	4.44	0.56	0.74	0.19	*
G6	wl	a	7	6.17	0.77	0.86	0.25	*
G6	dr	a	7	14.25	1.79	1.18	0.45	**
G1	ns	a	2	0.8	0.03	0.26	0.01	NA
G1	wl	a	2	0.13	0.01	0.05	0	NA
G1	dr	a	2	2.08	0.08	0.49	0.03	NA
G2	ns	a	2	4.81	0.19	0.76	0.08	*
G2	wl	a	2	1.38	0.05	0.38	0.02	NA
G2	dr	a	2	4.11	0.16	0.71	0.07	*
G3	ns	a	2	1.64	0.07	0.42	0.03	NA
G3	wl	a	2	0.14	0.01	0.06	0	NA
G3	dr	a	2	0.52	0.02	0.18	0.01	NA
G4	ns	a	2	0.09	0	0.04	0	NA
G4	wl	a	2	0.08	0	0.03	0	NA
G4	dr	a	2	0.25	0.01	0.1	0	NA
G5	ns	a	2	1.42	0.06	0.38	0.02	NA
G5	wl	a	2	0.1	0	0.04	0	NA
G5	dr	a	2	2.3	0.09	0.52	0.04	NA
G6	ns	a	2	0.72	0.03	0.23	0.01	NA
G6	wl	a	2	0.98	0.04	0.3	0.02	NA
G6	dr	a	2	0.88	0.03	0.27	0.01	NA
G1	ns	a	5	9.2	0.61	1.01	0.21	*
G1	wl	a	5	4.31	0.29	0.73	0.11	*
G1	dr	a	5	20.41	1.36	1.33	0.37	**
G2	ns	a	5	169.23	11.24	2.23	1.09	***
G2	wl	a	5	132.52	8.81	2.13	0.99	**
G2	dr	a	5	286.08	19.01	2.46	1.3	***
G3	ns	a	5	432.26	28.72	2.64	1.47	***
G3	wl	a	5	253.26	16.83	2.41	1.25	***

G3	dr	a	5	335.54	22.29	2.53	1.37	***
G4	ns	a	5	206.44	13.72	2.32	1.17	***
G4	wl	a	5	203.82	13.54	2.31	1.16	***
G4	dr	a	5	196.78	13.07	2.3	1.15	***
G5	ns	a	5	157.85	10.49	2.2	1.06	***
G5	wl	a	5	187.35	12.45	2.27	1.13	***
G5	dr	a	5	320.24	21.28	2.51	1.35	***
G6	ns	a	5	119.94	7.97	2.08	0.95	**
G6	wl	a	5	143.15	9.51	2.16	1.02	**
G6	dr	a	5	147.27	9.79	2.17	1.03	**
G1	ns	a	8	6.91	0.89	0.9	0.28	*
G1	wl	a	8	1.68	0.22	0.43	0.08	*
G1	dr	a	8	28.89	3.7	1.48	0.67	**
G2	ns	a	8	28.77	3.68	1.47	0.67	**
G2	wl	a	8	14.81	1.9	1.2	0.46	**
G2	dr	a	8	48.26	6.18	1.69	0.86	**
G3	ns	a	8	17.19	2.2	1.26	0.51	**
G3	wl	a	8	2.25	0.29	0.51	0.11	*
G3	dr	a	8	10.5	1.34	1.06	0.37	**
G4	ns	a	8	3.56	0.46	0.66	0.16	*
G4	wl	a	8	3.47	0.44	0.65	0.16	*
G4	dr	a	8	9.22	1.18	1.01	0.34	**
G5	ns	a	8	45.23	5.79	1.66	0.83	**
G5	wl	a	8	21.48	2.75	1.35	0.57	**
G5	dr	a	8	48.24	6.18	1.69	0.86	**
G6	ns	a	8	6.88	0.88	0.9	0.27	*
G6	wl	a	8	6.66	0.85	0.88	0.27	*
G6	dr	a	8	17.9	2.29	1.28	0.52	**
G1	ns	a	10	6.85	0.78	0.89	0.25	*
G1	wl	a	10	9.99	1.14	1.04	0.33	**
G1	dr	a	10	18.41	2.09	1.29	0.49	**
G2	ns	a	10	90.36	10.28	1.96	1.05	***

G2	wl	a	10	79.74	9.07	1.91	1	**
G2	dr	a	10	135.78	15.45	2.14	1.22	***
G3	ns	a	10	56.85	6.47	1.76	0.87	**
G3	wl	a	10	13.69	1.56	1.17	0.41	**
G3	dr	a	10	120.15	13.67	2.08	1.17	***
G4	ns	a	10	71.57	8.14	1.86	0.96	**
G4	wl	a	10	12.24	1.39	1.12	0.38	**
G4	dr	a	10	29.1	3.31	1.48	0.63	**
G5	ns	a	10	0.62	0.07	0.21	0.03	NA
G5	wl	a	10	1.11	0.13	0.32	0.05	*
G5	dr	a	10	9.53	1.08	1.02	0.32	**
G6	ns	a	10	22.57	2.57	1.37	0.55	**
G6	wl	a	10	42.15	4.8	1.64	0.76	**
G6	dr	a	10	42.16	4.8	1.64	0.76	**
G1	ns	a	13	1.8	0.03	0.45	0.01	NA
G1	wl	a	13	19.56	0.32	1.31	0.12	*
G1	dr	a	13	11.44	0.19	1.09	0.08	*
G2	ns	a	13	96.58	1.6	1.99	0.41	**
G2	wl	a	13	92.3	1.53	1.97	0.4	**
G2	dr	a	13	72.43	1.2	1.87	0.34	**
G3	ns	a	13	31.88	0.53	1.52	0.18	*
G3	wl	a	13	33.25	0.55	1.53	0.19	*
G3	dr	a	13	24.31	0.4	1.4	0.15	*
G4	ns	a	13	19.47	0.32	1.31	0.12	*
G4	wl	a	13	35.96	0.59	1.57	0.2	*
G4	dr	a	13	18.88	0.31	1.3	0.12	*
G5	ns	a	13	44.58	0.74	1.66	0.24	*
G5	wl	a	13	121.46	2.01	2.09	0.48	**
G5	dr	a	13	75.26	1.24	1.88	0.35	**
G6	ns	a	13	7.66	0.13	0.94	0.05	*
G6	wl	a	13	48.68	0.8	1.7	0.26	*
G6	dr	a	13	15.9	0.26	1.23	0.1	*

G1	ns	b	3	2.7	0.47	0.57	0.17	*
G1	wl	b	3	0.37	0.06	0.14	0.03	NA
G1	dr	b	3	4.65	0.82	0.75	0.26	*
G2	ns	b	3	1.33	0.23	0.37	0.09	*
G2	wl	b	3	0.29	0.05	0.11	0.02	NA
G2	dr	b	3	2.61	0.46	0.56	0.16	*
G3	ns	b	3	1.46	0.26	0.39	0.1	*
G3	wl	b	3	1.22	0.21	0.35	0.08	*
G3	dr	b	3	1.11	0.19	0.32	0.08	*
G4	ns	b	3	1.6	0.28	0.42	0.11	*
G4	wl	b	3	0.69	0.12	0.23	0.05	*
G4	dr	b	3	2.16	0.38	0.5	0.14	*
G5	ns	b	3	5.72	1	0.83	0.3	**
G5	wl	b	3	2.42	0.42	0.53	0.15	*
G5	dr	b	3	1.46	0.26	0.39	0.1	*
G6	ns	b	3	0.4	0.07	0.15	0.03	NA
G6	wl	b	3	0.13	0.02	0.05	0.01	NA
G6	dr	b	3	0.27	0.05	0.1	0.02	NA
G1	ns	b	1	0.01	0	0.01	0	NA
G1	wl	b	1	0.09	0.02	0.04	0.01	NA
G1	dr	b	1	0.05	0.01	0.02	0.01	NA
G2	ns	b	1	0.02	0.01	0.01	0	NA
G2	wl	b	1	0.91	0.22	0.28	0.09	*
G2	dr	b	1	0.02	0	0.01	0	NA
G3	ns	b	1	0.01	0	0	0	NA
G3	wl	b	1	1.89	0.45	0.46	0.16	*
G3	dr	b	1	0.45	0.11	0.16	0.04	*
G4	ns	b	1	0.01	0	0	0	NA
G4	wl	b	1	0.11	0.03	0.05	0.01	NA
G4	dr	b	1	0	0	0	0	NA
G5	ns	b	1	13.39	3.22	1.16	0.62	**
G5	wl	b	1	5.59	1.34	0.82	0.37	**

G5	dr	b	1	0.16	0.04	0.06	0.02	NA
G6	ns	b	1	0.01	0	0.01	0	NA
G6	wl	b	1	3.23	0.78	0.63	0.25	*
G6	dr	b	1	0.01	0	0	0	NA
G1	ns	b	11	0.51	0.03	0.18	0.01	NA
G1	wl	b	11	0.18	0.01	0.07	0	NA
G1	dr	b	11	0.49	0.02	0.17	0.01	NA
G2	ns	b	11	12.9	0.66	1.14	0.22	*
G2	wl	b	11	10.74	0.55	1.07	0.19	*
G2	dr	b	11	27.53	1.4	1.46	0.38	**
G3	ns	b	11	7.81	0.4	0.94	0.15	*
G3	wl	b	11	0.87	0.04	0.27	0.02	NA
G3	dr	b	11	3.23	0.16	0.63	0.07	*
G4	ns	b	11	3.83	0.19	0.68	0.08	*
G4	wl	b	11	2.15	0.11	0.5	0.05	*
G4	dr	b	11	14.67	0.75	1.2	0.24	*
G5	ns	b	11	0.07	0	0.03	0	NA
G5	wl	b	11	0.04	0	0.02	0	NA
G5	dr	b	11	0.07	0	0.03	0	NA
G6	ns	b	11	3.8	0.19	0.68	0.08	*
G6	wl	b	11	1.8	0.09	0.45	0.04	NA
G6	dr	b	11	3.44	0.17	0.65	0.07	*
G1	ns	b	14	270.47	76.72	2.43	1.89	***
G1	wl	b	14	224.05	63.56	2.35	1.81	***
G1	dr	b	14	458.8	130.15	2.66	2.12	***
G2	ns	b	14	608	172.47	2.78	2.24	***
G2	wl	b	14	230.36	65.35	2.36	1.82	***
G2	dr	b	14	1485.42	421.37	3.17	2.63	***
G3	ns	b	14	2298.19	651.94	3.36	2.81	***
G3	wl	b	14	1367.05	387.8	3.14	2.59	***
G3	dr	b	14	4705.49	1334.83	3.67	3.13	***
G4	ns	b	14	94.53	26.82	1.98	1.44	***

G4	wl	b	14	29.71	8.43	1.49	0.97	**
G4	dr	b	14	736.77	209	2.87	2.32	***
G5	ns	b	14	174.63	49.54	2.24	1.7	***
G5	wl	b	14	278.05	78.88	2.45	1.9	***
G5	dr	b	14	341.7	96.93	2.53	1.99	***
G6	ns	b	14	369.73	104.88	2.57	2.02	***
G6	wl	b	14	140.27	39.79	2.15	1.61	***
G6	dr	b	14	1517.14	430.37	3.18	2.63	***
G1	ns	b	12	5.12	0.45	0.79	0.16	*
G1	wl	b	12	1.19	0.11	0.34	0.04	*
G1	dr	b	12	7.16	0.63	0.91	0.21	*
G2	ns	b	12	6.99	0.62	0.9	0.21	*
G2	wl	b	12	4.54	0.4	0.74	0.15	*
G2	dr	b	12	15.03	1.33	1.21	0.37	**
G3	ns	b	12	12.55	1.11	1.13	0.32	**
G3	wl	b	12	6.38	0.56	0.87	0.19	*
G3	dr	b	12	11.52	1.02	1.1	0.31	**
G4	ns	b	12	2.38	0.21	0.53	0.08	*
G4	wl	b	12	0.92	0.08	0.28	0.03	NA
G4	dr	b	12	7.99	0.71	0.95	0.23	*
G5	ns	b	12	5.61	0.5	0.82	0.18	*
G5	wl	b	12	4.52	0.4	0.74	0.15	*
G5	dr	b	12	3.28	0.29	0.63	0.11	*
G6	ns	b	12	4.05	0.36	0.7	0.13	*
G6	wl	b	12	1.21	0.11	0.34	0.04	*
G6	dr	b	12	6.61	0.59	0.88	0.2	*
G1	ns	b	9	0.17	0.05	0.07	0.02	NA
G1	wl	b	9	0.09	0.03	0.04	0.01	NA
G1	dr	b	9	0.13	0.04	0.05	0.02	NA
G2	ns	b	9	17.26	5.15	1.26	0.79	**
G2	wl	b	9	15.68	4.68	1.22	0.75	**
G2	dr	b	9	30.01	8.96	1.49	1	**

G3	ns	b	9	5.37	1.6	0.8	0.42	**
G3	wl	b	9	0.12	0.04	0.05	0.02	NA
G3	dr	b	9	0.84	0.25	0.26	0.1	*
G4	ns	b	9	5.51	1.65	0.81	0.42	**
G4	wl	b	9	3.45	1.03	0.65	0.31	**
G4	dr	b	9	20.3	6.06	1.33	0.85	**
G5	ns	b	9	0.03	0.01	0.01	0	NA
G5	wl	b	9	0.01	0	0	0	NA
G5	dr	b	9	0.01	0	0	0	NA
G6	ns	b	9	7.09	2.12	0.91	0.49	**
G6	wl	b	9	5.57	1.66	0.82	0.43	**
G6	dr	b	9	3.8	1.14	0.68	0.33	**
G1	ns	b	6	4.37	0.42	0.73	0.15	*
G1	wl	b	6	2.18	0.21	0.5	0.08	*
G1	dr	b	6	5.9	0.57	0.84	0.2	*
G2	ns	b	6	118.53	11.38	2.08	1.09	***
G2	wl	b	6	104.06	9.99	2.02	1.04	**
G2	dr	b	6	206.88	19.86	2.32	1.32	***
G3	ns	b	6	70.49	6.77	1.85	0.89	**
G3	wl	b	6	68.18	6.55	1.84	0.88	**
G3	dr	b	6	84.58	8.12	1.93	0.96	**
G4	ns	b	6	26.26	2.52	1.44	0.55	**
G4	wl	b	6	33.31	3.2	1.54	0.62	**
G4	dr	b	6	62.15	5.97	1.8	0.84	**
G5	ns	b	6	130.85	12.56	2.12	1.13	***
G5	wl	b	6	115.87	11.13	2.07	1.08	***
G5	dr	b	6	67.71	6.5	1.84	0.88	**
G6	ns	b	6	50.7	4.87	1.71	0.77	**
G6	wl	b	6	22.63	2.17	1.37	0.5	**
G6	dr	b	6	71.85	6.9	1.86	0.9	**
G1	ns	b	4	1.89	0.22	0.46	0.09	*
G1	wl	b	4	0.67	0.08	0.22	0.03	NA

G1	dr	b	4	4.99	0.57	0.78	0.2	*
G2	ns	b	4	17.01	1.96	1.26	0.47	**
G2	wl	b	4	12.57	1.45	1.13	0.39	**
G2	dr	b	4	32.8	3.77	1.53	0.68	**
G3	ns	b	4	52.41	6.03	1.73	0.85	**
G3	wl	b	4	9.68	1.11	1.03	0.33	**
G3	dr	b	4	23.85	2.74	1.4	0.57	**
G4	ns	b	4	48.79	5.61	1.7	0.82	**
G4	wl	b	4	41.64	4.79	1.63	0.76	**
G4	dr	b	4	83.78	9.63	1.93	1.03	**
G5	ns	b	4	4.46	0.51	0.74	0.18	*
G5	wl	b	4	NA	NA	NA	NA	NA
G5	dr	b	4	4.63	0.53	0.75	0.19	*
G6	ns	b	4	76.38	8.78	1.89	0.99	**
G6	wl	b	4	83.27	9.58	1.93	1.02	**
G6	dr	b	4	60.56	6.96	1.79	0.9	**
G1	ns	b	7	9.44	1.19	1.02	0.34	**
G1	wl	b	7	1.27	0.16	0.36	0.06	*
G1	dr	b	7	22.59	2.84	1.37	0.58	**
G2	ns	b	7	20.28	2.55	1.33	0.55	**
G2	wl	b	7	5.86	0.74	0.84	0.24	*
G2	dr	b	7	35.3	4.43	1.56	0.73	**
G3	ns	b	7	20.85	2.62	1.34	0.56	**
G3	wl	b	7	4.09	0.51	0.71	0.18	*
G3	dr	b	7	14.3	1.8	1.18	0.45	**
G4	ns	b	7	10.19	1.28	1.05	0.36	**
G4	wl	b	7	3.86	0.48	0.69	0.17	*
G4	dr	b	7	13.57	1.7	1.16	0.43	**
G5	ns	b	7	5.97	0.75	0.84	0.24	*
G5	wl	b	7	2.12	0.27	0.49	0.1	*
G5	dr	b	7	3.55	0.45	0.66	0.16	*
G6	ns	b	7	7.01	0.88	0.9	0.27	*

G6	wl	b	7	3.05	0.38	0.61	0.14	*
G6	dr	b	7	18.91	2.37	1.3	0.53	**
G1	ns	b	2	1.35	0.05	0.37	0.02	NA
G1	wl	b	2	0.2	0.01	0.08	0	NA
G1	dr	b	2	2.29	0.09	0.52	0.04	NA
G2	ns	b	2	3.64	0.15	0.67	0.06	*
G2	wl	b	2	1.09	0.04	0.32	0.02	NA
G2	dr	b	2	7.75	0.31	0.94	0.12	*
G3	ns	b	2	0.25	0.01	0.1	0	NA
G3	wl	b	2	0.28	0.01	0.11	0	NA
G3	dr	b	2	0.15	0.01	0.06	0	NA
G4	ns	b	2	0.17	0.01	0.07	0	NA
G4	wl	b	2	0.07	0	0.03	0	NA
G4	dr	b	2	0.32	0.01	0.12	0.01	NA
G5	ns	b	2	1.9	0.08	0.46	0.03	NA
G5	wl	b	2	0.85	0.03	0.27	0.01	NA
G5	dr	b	2	0.26	0.01	0.1	0	NA
G6	ns	b	2	6.83	0.27	0.89	0.1	*
G6	wl	b	2	0.45	0.02	0.16	0.01	NA
G6	dr	b	2	0.57	0.02	0.19	0.01	NA
G1	ns	b	5	9.6	0.64	1.03	0.21	*
G1	wl	b	5	5.63	0.37	0.82	0.14	*
G1	dr	b	5	53.22	3.54	1.73	0.66	**
G2	ns	b	5	161.24	10.71	2.21	1.07	***
G2	wl	b	5	84.35	5.6	1.93	0.82	**
G2	dr	b	5	262.61	17.45	2.42	1.27	***
G3	ns	b	5	179.78	11.94	2.26	1.11	***
G3	wl	b	5	122.13	8.11	2.09	0.96	**
G3	dr	b	5	378.27	25.13	2.58	1.42	***
G4	ns	b	5	171.14	11.37	2.24	1.09	***
G4	wl	b	5	105.38	7	2.03	0.9	**
G4	dr	b	5	303.36	20.16	2.48	1.33	***

G5	ns	b	5	434.79	28.89	2.64	1.48	***
G5	wl	b	5	351.51	23.36	2.55	1.39	***
G5	dr	b	5	222.44	14.78	2.35	1.2	***
G6	ns	b	5	173.31	11.52	2.24	1.1	***
G6	wl	b	5	142.65	9.48	2.16	1.02	**
G6	dr	b	5	227.64	15.13	2.36	1.21	***
G1	ns	b	8	24.71	3.16	1.41	0.62	**
G1	wl	b	8	1.98	0.25	0.47	0.1	*
G1	dr	b	8	31.74	4.06	1.52	0.7	**
G2	ns	b	8	28.17	3.61	1.46	0.66	**
G2	wl	b	8	8.17	1.05	0.96	0.31	**
G2	dr	b	8	50.77	6.5	1.71	0.88	**
G3	ns	b	8	13.69	1.75	1.17	0.44	**
G3	wl	b	8	6.28	0.8	0.86	0.26	*
G3	dr	b	8	16.68	2.14	1.25	0.5	**
G4	ns	b	8	7.04	0.9	0.91	0.28	*
G4	wl	b	8	2.17	0.28	0.5	0.11	*
G4	dr	b	8	9.51	1.22	1.02	0.35	**
G5	ns	b	8	70.84	9.07	1.86	1	**
G5	wl	b	8	25.63	3.28	1.43	0.63	**
G5	dr	b	8	25.92	3.32	1.43	0.64	**
G6	ns	b	8	20.05	2.57	1.32	0.55	**
G6	wl	b	8	4.78	0.61	0.76	0.21	*
G6	dr	b	8	18.44	2.36	1.29	0.53	**
G1	ns	b	10	8.86	1.01	0.99	0.3	**
G1	wl	b	10	4.59	0.52	0.75	0.18	*
G1	dr	b	10	15.25	1.73	1.21	0.44	**
G2	ns	b	10	104.54	11.89	2.02	1.11	***
G2	wl	b	10	76.03	8.65	1.89	0.98	**
G2	dr	b	10	198.92	22.63	2.3	1.37	***
G3	ns	b	10	71.32	8.11	1.86	0.96	**
G3	wl	b	10	20.86	2.37	1.34	0.53	**

G3	dr	b	10	36.98	4.21	1.58	0.72	**
G4	ns	b	10	26.24	2.99	1.44	0.6	**
G4	wl	b	10	16.84	1.92	1.25	0.46	**
G4	dr	b	10	115.48	13.14	2.07	1.15	***
G5	ns	b	10	10.63	1.21	1.07	0.34	**
G5	wl	b	10	1.53	0.17	0.4	0.07	*
G5	dr	b	10	3.76	0.43	0.68	0.15	*
G6	ns	b	10	25.71	2.92	1.43	0.59	**
G6	wl	b	10	16.81	1.91	1.25	0.46	**
G6	dr	b	10	47.97	5.46	1.69	0.81	**
G1	ns	b	13	4.95	0.08	0.77	0.03	NA
G1	wl	b	13	8.79	0.15	0.99	0.06	*
G1	dr	b	13	13.57	0.22	1.16	0.09	*
G2	ns	b	13	75.79	1.25	1.89	0.35	**
G2	wl	b	13	94.6	1.56	1.98	0.41	**
G2	dr	b	13	153.11	2.53	2.19	0.55	**
G3	ns	b	13	31.04	0.51	1.51	0.18	*
G3	wl	b	13	44.63	0.74	1.66	0.24	*
G3	dr	b	13	32.89	0.54	1.53	0.19	*
G4	ns	b	13	16.62	0.27	1.25	0.11	*
G4	wl	b	13	25.56	0.42	1.42	0.15	*
G4	dr	b	13	14.9	0.25	1.2	0.1	*
G5	ns	b	13	75.1	1.24	1.88	0.35	**
G5	wl	b	13	84.39	1.4	1.93	0.38	**
G5	dr	b	13	76.95	1.27	1.89	0.36	**
G6	ns	b	13	23.49	0.39	1.39	0.14	*
G6	wl	b	13	17.99	0.3	1.28	0.11	*
G6	dr	b	13	27.1	0.45	1.45	0.16	*
G1	ns	c	3	0.95	0.17	0.29	0.07	*
G1	wl	c	3	1.22	0.21	0.35	0.08	*
G1	dr	c	3	3.17	0.56	0.62	0.19	*
G2	ns	c	3	3.55	0.62	0.66	0.21	*

G2	wl	c	3	0.23	0.04	0.09	0.02	NA
G2	dr	c	3	2.59	0.46	0.56	0.16	*
G3	ns	c	3	1.4	0.25	0.38	0.1	*
G3	wl	c	3	0.76	0.13	0.24	0.05	*
G3	dr	c	3	5.42	0.95	0.81	0.29	*
G4	ns	c	3	1.78	0.31	0.44	0.12	*
G4	wl	c	3	0.56	0.1	0.19	0.04	NA
G4	dr	c	3	1.31	0.23	0.36	0.09	*
G5	ns	c	3	4.15	0.73	0.71	0.24	*
G5	wl	c	3	0.98	0.17	0.3	0.07	*
G5	dr	c	3	3.1	0.54	0.61	0.19	*
G6	ns	c	3	0.04	0.01	0.02	0	NA
G6	wl	c	3	0.29	0.05	0.11	0.02	NA
G6	dr	c	3	0.33	0.06	0.12	0.02	NA
G1	ns	c	1	0.02	0	0.01	0	NA
G1	wl	c	1	6.61	1.59	0.88	0.41	**
G1	dr	c	1	0.15	0.04	0.06	0.02	NA
G2	ns	c	1	0.02	0.01	0.01	0	NA
G2	wl	c	1	0.35	0.08	0.13	0.03	NA
G2	dr	c	1	0.01	0	0	0	NA
G3	ns	c	1	0.02	0.01	0.01	0	NA
G3	wl	c	1	2.77	0.67	0.58	0.22	*
G3	dr	c	1	0.02	0.01	0.01	0	NA
G4	ns	c	1	0.02	0	0.01	0	NA
G4	wl	c	1	0.09	0.02	0.04	0.01	NA
G4	dr	c	1	0.01	0	0	0	NA
G5	ns	c	1	0.02	0	0.01	0	NA
G5	wl	c	1	3.4	0.82	0.64	0.26	*
G5	dr	c	1	0.14	0.03	0.06	0.01	NA
G6	ns	c	1	0.05	0.01	0.02	0	NA
G6	wl	c	1	0.87	0.21	0.27	0.08	*
G6	dr	c	1	0.01	0	0	0	NA

G1	ns	c	11	1.82	0.09	0.45	0.04	NA
G1	wl	c	11	0.37	0.02	0.14	0.01	NA
G1	dr	c	11	0.56	0.03	0.19	0.01	NA
G2	ns	c	11	29	1.47	1.48	0.39	**
G2	wl	c	11	7.16	0.36	0.91	0.13	*
G2	dr	c	11	25.7	1.31	1.43	0.36	**
G3	ns	c	11	1.46	0.07	0.39	0.03	NA
G3	wl	c	11	3.33	0.17	0.64	0.07	*
G3	dr	c	11	3.29	0.17	0.63	0.07	*
G4	ns	c	11	10.6	0.54	1.06	0.19	*
G4	wl	c	11	1.62	0.08	0.42	0.03	NA
G4	dr	c	11	5.54	0.28	0.82	0.11	*
G5	ns	c	11	0.08	0	0.03	0	NA
G5	wl	c	11	0.18	0.01	0.07	0	NA
G5	dr	c	11	0.45	0.02	0.16	0.01	NA
G6	ns	c	11	2.91	0.15	0.59	0.06	*
G6	wl	c	11	1.7	0.09	0.43	0.04	NA
G6	dr	c	11	1.5	0.08	0.4	0.03	NA
G1	ns	c	14	1297.43	368.05	3.11	2.57	***
G1	wl	c	14	1026.43	291.17	3.01	2.47	***
G1	dr	c	14	1084.76	307.72	3.04	2.49	***
G2	ns	c	14	937.91	266.06	2.97	2.43	***
G2	wl	c	14	279.2	79.2	2.45	1.9	***
G2	dr	c	14	1566.81	444.46	3.2	2.65	***
G3	ns	c	14	1823.05	517.15	3.26	2.71	***
G3	wl	c	14	3092.87	877.37	3.49	2.94	***
G3	dr	c	14	2022.72	573.79	3.31	2.76	***
G4	ns	c	14	410.19	116.36	2.61	2.07	***
G4	wl	c	14	26.91	7.63	1.45	0.94	**
G4	dr	c	14	217.37	61.66	2.34	1.8	***
G5	ns	c	14	536.65	152.23	2.73	2.19	***
G5	wl	c	14	264.5	75.03	2.42	1.88	***

G5	dr	c	14	954.53	270.77	2.98	2.43	***
G6	ns	c	14	539.01	152.9	2.73	2.19	***
G6	wl	c	14	149.03	42.27	2.18	1.64	***
G6	dr	c	14	175.47	49.78	2.25	1.71	***
G1	ns	c	12	7.96	0.7	0.95	0.23	*
G1	wl	c	12	5	0.44	0.78	0.16	*
G1	dr	c	12	7.2	0.64	0.91	0.21	*
G2	ns	c	12	12.45	1.1	1.13	0.32	**
G2	wl	c	12	2.86	0.25	0.59	0.1	*
G2	dr	c	12	13.36	1.18	1.16	0.34	**
G3	ns	c	12	9.57	0.85	1.02	0.27	*
G3	wl	c	12	13.88	1.23	1.17	0.35	**
G3	dr	c	12	13.37	1.18	1.16	0.34	**
G4	ns	c	12	6.92	0.61	0.9	0.21	*
G4	wl	c	12	0.77	0.07	0.25	0.03	NA
G4	dr	c	12	3.5	0.31	0.65	0.12	*
G5	ns	c	12	4.87	0.43	0.77	0.16	*
G5	wl	c	12	6.67	0.59	0.88	0.2	*
G5	dr	c	12	6.59	0.58	0.88	0.2	*
G6	ns	c	12	1.95	0.17	0.47	0.07	*
G6	wl	c	12	1.87	0.17	0.46	0.07	*
G6	dr	c	12	1.41	0.12	0.38	0.05	*
G1	ns	c	9	1.84	0.55	0.45	0.19	*
G1	wl	c	9	0.04	0.01	0.02	0	NA
G1	dr	c	9	0.14	0.04	0.06	0.02	NA
G2	ns	c	9	34.17	10.2	1.55	1.05	***
G2	wl	c	9	12.42	3.71	1.13	0.67	**
G2	dr	c	9	32.05	9.57	1.52	1.02	**
G3	ns	c	9	0.19	0.06	0.08	0.02	NA
G3	wl	c	9	0.33	0.1	0.12	0.04	NA
G3	dr	c	9	0.64	0.19	0.22	0.08	*
G4	ns	c	9	13.69	4.09	1.17	0.71	**

G4	wl	c	9	2.5	0.75	0.54	0.24	*
G4	dr	c	9	7.26	2.17	0.92	0.5	**
G5	ns	c	9	0.13	0.04	0.05	0.02	NA
G5	wl	c	9	0.01	0	0	0	NA
G5	dr	c	9	0.03	0.01	0.01	0	NA
G6	ns	c	9	4.08	1.22	0.71	0.35	**
G6	wl	c	9	3.28	0.98	0.63	0.3	*
G6	dr	c	9	2.88	0.86	0.59	0.27	*
G1	ns	c	6	40.12	3.85	1.61	0.69	**
G1	wl	c	6	59.58	5.72	1.78	0.83	**
G1	dr	c	6	29.71	2.85	1.49	0.59	**
G2	ns	c	6	208.23	19.99	2.32	1.32	***
G2	wl	c	6	112.34	10.79	2.05	1.07	***
G2	dr	c	6	281.77	27.05	2.45	1.45	***
G3	ns	c	6	19.81	1.9	1.32	0.46	**
G3	wl	c	6	142.17	13.65	2.16	1.17	***
G3	dr	c	6	45.1	4.33	1.66	0.73	**
G4	ns	c	6	95.63	9.18	1.99	1.01	**
G4	wl	c	6	28.07	2.7	1.46	0.57	**
G4	dr	c	6	30.35	2.91	1.5	0.59	**
G5	ns	c	6	89.35	8.58	1.96	0.98	**
G5	wl	c	6	133.98	12.86	2.13	1.14	***
G5	dr	c	6	113.64	10.91	2.06	1.08	***
G6	ns	c	6	33.08	3.18	1.53	0.62	**
G6	wl	c	6	35.25	3.38	1.56	0.64	**
G6	dr	c	6	23.68	2.27	1.39	0.52	**
G1	ns	c	4	6.44	0.74	0.87	0.24	*
G1	wl	c	4	3.55	0.41	0.66	0.15	*
G1	dr	c	4	4.38	0.5	0.73	0.18	*
G2	ns	c	4	40.37	4.64	1.62	0.75	**
G2	wl	c	4	7.05	0.81	0.91	0.26	*
G2	dr	c	4	29	3.33	1.48	0.64	**

G3	ns	c	4	11.32	1.3	1.09	0.36	**
G3	wl	c	4	17.99	2.07	1.28	0.49	**
G3	dr	c	4	62.06	7.14	1.8	0.91	**
G4	ns	c	4	80.15	9.22	1.91	1.01	**
G4	wl	c	4	23.07	2.65	1.38	0.56	**
G4	dr	c	4	58.89	6.77	1.78	0.89	**
G5	ns	c	4	NA	NA	NA	NA	NA
G5	wl	c	4	6.16	0.71	0.85	0.23	*
G5	dr	c	4	9.96	1.15	1.04	0.33	**
G6	ns	c	4	26.76	3.08	1.44	0.61	**
G6	wl	c	4	18.61	2.14	1.29	0.5	**
G6	dr	c	4	21.56	2.48	1.35	0.54	**
G1	ns	c	7	4.21	0.53	0.72	0.18	*
G1	wl	c	7	2.55	0.32	0.55	0.12	*
G1	dr	c	7	11.61	1.46	1.1	0.39	**
G2	ns	c	7	28.99	3.64	1.48	0.67	**
G2	wl	c	7	8.42	1.06	0.97	0.31	**
G2	dr	c	7	32.49	4.08	1.52	0.71	**
G3	ns	c	7	9.94	1.25	1.04	0.35	**
G3	wl	c	7	8.77	1.1	0.99	0.32	**
G3	dr	c	7	11.36	1.43	1.09	0.38	**
G4	ns	c	7	10.94	1.37	1.08	0.38	**
G4	wl	c	7	2.99	0.38	0.6	0.14	*
G4	dr	c	7	8.24	1.03	0.97	0.31	**
G5	ns	c	7	5.93	0.74	0.84	0.24	*
G5	wl	c	7	1.87	0.23	0.46	0.09	*
G5	dr	c	7	8.91	1.12	1	0.33	**
G6	ns	c	7	2.15	0.27	0.5	0.1	*
G6	wl	c	7	6.2	0.78	0.86	0.25	*
G6	dr	c	7	2.95	0.37	0.6	0.14	*
G1	ns	c	2	0.6	0.02	0.2	0.01	NA
G1	wl	c	2	0.63	0.02	0.21	0.01	NA

G1	dr	c	2	1.63	0.06	0.42	0.03	NA
G2	ns	c	2	9.53	0.38	1.02	0.14	*
G2	wl	c	2	1.2	0.05	0.34	0.02	NA
G2	dr	c	2	6.44	0.26	0.87	0.1	*
G3	ns	c	2	0.4	0.02	0.15	0.01	NA
G3	wl	c	2	0.71	0.03	0.23	0.01	NA
G3	dr	c	2	1.12	0.04	0.33	0.02	NA
G4	ns	c	2	0.3	0.01	0.11	0.01	NA
G4	wl	c	2	0.06	0	0.02	0	NA
G4	dr	c	2	0.13	0.01	0.05	0	NA
G5	ns	c	2	2.31	0.09	0.52	0.04	NA
G5	wl	c	2	0.14	0.01	0.06	0	NA
G5	dr	c	2	0.77	0.03	0.25	0.01	NA
G6	ns	c	2	0.87	0.03	0.27	0.01	NA
G6	wl	c	2	0.18	0.01	0.07	0	NA
G6	dr	c	2	0.12	0	0.05	0	NA
G1	ns	c	5	29.4	1.95	1.48	0.47	**
G1	wl	c	5	21.64	1.44	1.35	0.39	**
G1	dr	c	5	21.21	1.41	1.35	0.38	**
G2	ns	c	5	317.87	21.12	2.5	1.34	***
G2	wl	c	5	103.63	6.89	2.02	0.9	**
G2	dr	c	5	397.86	26.43	2.6	1.44	***
G3	ns	c	5	188.89	12.55	2.28	1.13	***
G3	wl	c	5	281.59	18.71	2.45	1.29	***
G3	dr	c	5	372.49	24.75	2.57	1.41	***
G4	ns	c	5	387.1	25.72	2.59	1.43	***
G4	wl	c	5	104.23	6.93	2.02	0.9	**
G4	dr	c	5	206.44	13.72	2.32	1.17	***
G5	ns	c	5	345.26	22.94	2.54	1.38	***
G5	wl	c	5	367.41	24.41	2.57	1.41	***
G5	dr	c	5	369.97	24.58	2.57	1.41	***
G6	ns	c	5	97.84	6.5	1.99	0.88	**

G6	wl	c	5	88.53	5.88	1.95	0.84	**
G6	dr	c	5	81.57	5.42	1.92	0.81	**
G1	ns	c	8	6.85	0.88	0.89	0.27	*
G1	wl	c	8	5.69	0.73	0.83	0.24	*
G1	dr	c	8	21.25	2.72	1.35	0.57	**
G2	ns	c	8	65.18	8.35	1.82	0.97	**
G2	wl	c	8	14.02	1.8	1.18	0.45	**
G2	dr	c	8	53.89	6.9	1.74	0.9	**
G3	ns	c	8	15.86	2.03	1.23	0.48	**
G3	wl	c	8	6.7	0.86	0.89	0.27	*
G3	dr	c	8	47.6	6.1	1.69	0.85	**
G4	ns	c	8	7.56	0.97	0.93	0.29	*
G4	wl	c	8	2.01	0.26	0.48	0.1	*
G4	dr	c	8	5.86	0.75	0.84	0.24	*
G5	ns	c	8	47.43	6.07	1.69	0.85	**
G5	wl	c	8	23.81	3.05	1.39	0.61	**
G5	dr	c	8	41.11	5.26	1.62	0.8	**
G6	ns	c	8	4.74	0.61	0.76	0.21	*
G6	wl	c	8	5.19	0.67	0.79	0.22	*
G6	dr	c	8	1.64	0.21	0.42	0.08	*
G1	ns	c	10	26.89	3.06	1.45	0.61	**
G1	wl	c	10	11.83	1.35	1.11	0.37	**
G1	dr	c	10	20.88	2.38	1.34	0.53	**
G2	ns	c	10	206.27	23.47	2.32	1.39	***
G2	wl	c	10	55.65	6.33	1.75	0.87	**
G2	dr	c	10	187.05	21.28	2.27	1.35	***
G3	ns	c	10	19.58	2.23	1.31	0.51	**
G3	wl	c	10	46.18	5.25	1.67	0.8	**
G3	dr	c	10	53.93	6.14	1.74	0.85	**
G4	ns	c	10	91	10.35	1.96	1.06	***
G4	wl	c	10	12.14	1.38	1.12	0.38	**
G4	dr	c	10	42.22	4.8	1.64	0.76	**

G5	ns	c	10	1.47	0.17	0.39	0.07	*
G5	wl	c	10	2.34	0.27	0.52	0.1	*
G5	dr	c	10	10.69	1.22	1.07	0.35	**
G6	ns	c	10	32.36	3.68	1.52	0.67	**
G6	wl	c	10	17.17	1.95	1.26	0.47	**
G6	dr	c	10	25.61	2.91	1.43	0.59	**
G1	ns	c	13	21.61	0.36	1.35	0.13	*
G1	wl	c	13	44.84	0.74	1.66	0.24	*
G1	dr	c	13	15.33	0.25	1.21	0.1	*
G2	ns	c	13	140.68	2.33	2.15	0.52	**
G2	wl	c	13	92.12	1.52	1.97	0.4	**
G2	dr	c	13	148.9	2.46	2.18	0.54	**
G3	ns	c	13	33.89	0.56	1.54	0.19	*
G3	wl	c	13	36.37	0.6	1.57	0.2	*
G3	dr	c	13	24.81	0.41	1.41	0.15	*
G4	ns	c	13	36.39	0.6	1.57	0.2	*
G4	wl	c	13	14.13	0.23	1.18	0.09	*
G4	dr	c	13	17.7	0.29	1.27	0.11	*
G5	ns	c	13	53.81	0.89	1.74	0.28	*
G5	wl	c	13	70.84	1.17	1.86	0.34	**
G5	dr	c	13	70.88	1.17	1.86	0.34	**
G6	ns	c	13	19.21	0.32	1.31	0.12	*
G6	wl	c	13	25.72	0.43	1.43	0.15	*
G6	dr	c	13	7.87	0.13	0.95	0.05	*

Table S5: Overall analysis using pair wise Wilcoxon-Mann-Whitney-Test to find significantly different metabolites across different experimental conditions.

Compounds	Group 1	Group 2	N1	N2	P	P.adj	P.adj.signif
7	dr	wl	6	6	0.004	0.013	*
1	dr	wl	6	6	0.041	0.123	ns
1	dr	ns	6	6	0.937	1	ns
1	wl	ns	6	6	0.026	0.078	ns
10	dr	wl	6	6	0.24	0.72	ns
10	dr	ns	6	6	0.589	1	ns
10	wl	ns	6	6	0.699	1	ns
11	dr	wl	6	6	0.24	0.72	ns
11	dr	ns	6	6	0.699	1	ns
11	wl	ns	6	6	0.485	1	ns
12	dr	wl	6	6	0.041	0.123	ns
12	dr	ns	6	6	0.31	0.93	ns
12	wl	ns	6	6	0.589	1	ns
13	dr	wl	6	6	0.18	0.54	ns
13	dr	ns	6	6	0.937	1	ns
13	wl	ns	6	6	0.18	0.54	ns
14	dr	wl	6	6	0.394	1	ns
14	dr	ns	6	6	0.394	1	ns
14	wl	ns	6	6	0.132	0.396	ns
2	dr	wl	6	6	0.065	0.195	ns
2	dr	ns	6	6	0.818	1	ns
2	wl	ns	6	6	0.18	0.54	ns
3	dr	wl	6	6	0.18	0.54	ns
3	dr	ns	6	6	0.937	1	ns
3	wl	ns	6	6	0.18	0.54	ns
4	dr	wl	6	6	0.24	0.72	ns
4	dr	ns	6	6	0.31	0.93	ns
4	wl	ns	6	6	0.818	1	ns
5	dr	wl	6	6	0.24	0.72	ns
5	dr	ns	6	6	0.589	1	ns

5	wl	ns	6	6	0.937	1	ns
6	dr	wl	6	6	0.589	1	ns
6	dr	ns	6	6	0.589	1	ns
6	wl	ns	6	6	0.24	0.72	ns
7	dr	ns	6	6	0.026	0.078	ns
7	wl	ns	6	6	0.24	0.72	ns
8	dr	wl	6	6	0.041	0.123	ns
8	dr	ns	6	6	0.18	0.54	ns
8	wl	ns	6	6	0.132	0.396	ns
9	dr	wl	6	6	0.589	1	ns
9	dr	ns	6	6	0.937	1	ns
9	wl	ns	6	6	0.485	1	ns

Table S6: The ANOVA and Tukey analyses showed differences between the cultivars (CV) and the different water supply (WSC) for the expression of the agronomical traits (bold letters)

ANOVA						
**PH**						
	SQ	FG	MQ	F	p	
Constant	225816.0	1	225816.0	16172.50	0.000000	
CV	3660.0	5	732.0	52.42	0.000000	sig
WSC	829.8	2	414.9	29.71	0.000000	sig
CV* WSC	157.6	10	15.8	1.13	0.369016	ns
Error	502.7	36	14.0			
**LM**						
	SQ	FG	MQ	F	p	
Constant	233438438	1	233438438	676.6631	0.000000	
CV	27313465	5	5462693	15.8346	0.000000	sig
WSC	32038900	2	16019450	46.4352	0.000000	sig
CV* WSC	4913422	10	491342	1.4242	0.209325	ns
Error	12419450	36	344985			
**MR-n**						
	SQ	FG	MQ	F	p	
Constant	58016.67	1	58016.67	1414.402	0.000000	
CV	2884.44	5	576.89	14.064	0.000000	sig
WSC	108.00	2	54.00	1.316	0.280674	ns
CV* WSC	324.22	10	32.42	0.790	0.637951	ns
Error	1476.67	36	41.02			
**MR-m**						
	SQ	FG	MQ	F	p	
Constant	448156838	1	448156838	1451.187	0.000000	
CV	33843638	5	6768728	21.918	0.000000	sig

WSC	7396836	2	3698418	11.976	0.000103	sig
CV* WSC	3717364	10	371736	1.204	0.321239	ns
Error	11117550	36	308821			

SUM-n

	SQ	FG	MQ	F	p	
Constant	59866.74	1	59866.74	1392.250	0.000000	
CV	3104.81	5	620.96	14.441	0.000000	sig
WSC	181.59	2	90.80	2.112	0.135796	ns
CV* WSC	320.85	10	32.09	0.746	0.677189	ns
Error	1548.00	36	43.00			

SUM-m

	SQ	FG	MQ	F	p	
Constant	462325556	1	462325556	1491.640	0.000000	
CV	34857780	5	6971556	22.493	0.000000	sig
WSC	8558473	2	4279237	13.806	0.000035	sig
CV* WSC	3764416	10	376442	1.215	0.314792	ns
Error	11158000	36	309944			

ALM

	SQ	FG	MQ	F	p	
Constant	281566.1	1	281566.1	129.3500	0.000000	
CV	112014.8	5	22403.0	10.2918	0.000003	sig
App	31500.5	2	15750.3	7.2356	0.002284	sig
CV* WSC	18816.3	10	1881.6	0.8644	0.573230	ns
Error	78363.9	36	2176.8			

ARM

	SQ	FG	MQ	F	p	
Constant	430296.8	1	430296.8	1231.736	0.000000	
CV	12128.8	5	2425.8	6.944	0.000125	sig

WSC	4262.2	2	2131.1	6.100	0.005230	sig
CV* WSC	452.5	10	45.3	0.130	0.999189	ns
Error	12576.3	36	349.3			

UR-n

	SQ	FG	MQ	F	p	
Constant	10113.35	1	10113.35	316.0422	0.000000	
CV	874.09	5	174.82	5.4631	0.000769	sig
WSC	281.04	2	140.52	4.3912	0.019657	sig
No* WSC	312.52	10	31.25	0.9766	0.480105	ns
Error	1152.00	36	32.00			

UR-m

	SQ	FG	MQ	F	p	
Constant	1277278	1	1277278	287.3876	0.000000	
CV	57097	5	11419	2.5694	0.043573	sig
WSC	25729	2	12864	2.8945	0.068287	ns
No* WSC	78321	10	7832	1.7622	0.104154	ns
Error	160000	36	4444			

Tukey

Tukey HSD Test; Variable **PH;** Alpha = 0.050; Error: MQ= 13.963; FG = 36

	No	CV	WSC	**PH (mean)**	1.	2	3	4	5	6	7	8	9
12	G4	ROS	dr	45.00	****								
11	G4	ROS	ns	53.00	****	****							
15	G5	GOS	dr	55.33	****	****	****						
10	G4	ROS	wl	55.67	****	****	****						
2	G1	TEX	ns	56.00	****	****	****						
3	G1	TEX	dr	58.67		****	****	****					

14	G5	GOS	ns	59.33	****	****	****	****				
18	G6	VIL	dr	61.67	****	****	****	****	****			
17	G6	VIL	ns	65.67		****	****	****	****	****		
1	G1	TEX	wl	66.00		****	****	****	****	****	****	
13	G5	GOS	wl	69.67			****	****	****	****	****	****
6	G2	NAG	dr	70.33				****	****	****	****	****
16	G6	VIL	wl	71.00					****	****	****	****
9	G3	SEN	dr	71.67					****	****	****	****
8	G3	SEN	ns	73.33						****	****	****
5	G2	NAG	ns	74.67						****	****	****
4	G2	NAG	wl	77.33							****	****
7	G3	SEN	wl	79.67								****

Tukey HSD Test; Variable **LM**; Alpha = 0.05; Error: MQ=3450E2; FG = 36

	No	CV	WSC	**LM (mean)**	1.	2	3	4	5	6
12	G4	ROS	dr	751.67	****					
11	G4	ROS	ns	816.67	****	****				
15	G5	GOS	dr	968.33	****	****				
3	G1	TEX	dr	1080.00	****	****				
10	G4	ROS	wl	1193.33	****	****	****			
2	G1	TEX	ns	1408.33	****	****	****			
18	G6	VIL	dr	1431.67	****	****	****			
14	G5	GOS	ns	1476.67	****	****	****			
17	G6	VIL	ns	1680.00	****	****	****			
6	G2	NAG	dr	1930.00	****	****	****	****		
9	G3	SEN	dr	2083.33	****	****	****	****	****	
5	G2	NAG	ns	2418.33	****	****	****	****	****	
8	G3	SEN	ns	2475.00	****	****	****	****	****	
13	G5	GOS	wl	2595.00		****	****	****	****	
1	G1	TEX	wl	2940.00			****	****	****	****

16	G6	VIL	wl	3636.67				****	****	****
4	G2	NAG	wl	3863.33					****	****
7	G3	SEN	wl	4676.67						****

Tukey HSD Test; Variable **MR-n;** Alpha =0.050; Error: MQ= 47.093; FG = 36

	No	CV	WSC	MR-n (mean)	1.	2	3	4
8	G3	SEN	ns	18.33	****			
9	G3	SEN	dr	19.67	****	****		
7	G3	SEN	wl	21.00	****	****	****	
5	G2	NAG	ns	27.67	****	****	****	****
15	G5	GOS	dr	28.33	****	****	****	****
6	G2	NAG	dr	29.00	****	****	****	****
17	G6	VIL	ns	30.33	****	****	****	****
4	G2	NAG	wl	31.00	****	****	****	****
11	G4	ROS	ns	31.67	****	****	****	****
10	G4	ROS	wl	32.67	****	****	****	****
18	G6	VIL	dr	35.00	****	****	****	****
12	G4	ROS	dr	36.00	****	****	****	****
13	G5	GOS	wl	38.67		****	****	****
14	G5	GOS	ns	39.33			****	****
16	G6	VIL	wl	39.67			****	****
3	G1	TEX	dr	42.67				****
2	G1	TEX	ns	43.33				****
1	G1	TEX	wl	45.67				****

Tukey HSD Test; Variable **MR-m;** Alpha =0.05; Error: MQ= 3249E2 FG = 36

	No	CV	WSC	MR-m (mean)	1.	2	3	4	5	6
9	G3	SEN	dr	1556.67	****					
8	G3	SEN	ns	1835.00	****	****				

15	G5	GOS	dr	1856.67	****	****				
6	G2	NAG	dr	2010.00	****	****	****			
7	G3	SEN	wl	2016.67	****	****	****			
5	G2	NAG	ns	2175.00	****	****	****	****		
4	G2	NAG	wl	2488.33	****	****	****	****	****	
12	G4	ROS	dr	2520.00	****	****	****	****	****	
11	G4	ROS	ns	2876.67	****	****	****	****	****	
10	G4	ROS	wl	2918.33	****	****	****	****	****	
14	G5	GOS	ns	2920.00	****	****	****	****	****	
3	G1	TEX	dr	2951.67	****	****	****	****	****	
13	G5	GOS	wl	3468.33		****	****	****	****	
17	G6	VIL	ns	3646.67			****	****	****	
18	G6	VIL	dr	3721.67				****	****	****
2	G1	TEX	ns	3731.67				****	****	****
1	G1	TEX	wl	4010.00					****	****
16	G6	VIL	wl	5151.67						****

Tukey HSD Test; Variable **ALM**; Alpha = 0.050; Error: MQ = 2176.8; FG = 36

	No	CV	WSC	**ALM**	1.	2
12	G4	ROS	dr	21.22	****	
3	G1	TEX	dr	25.34	****	
11	G4	ROS	ns	25.38	****	
15	G5	GOS	dr	31.95	****	
2	G1	TEX	ns	33.13	****	
14	G5	GOS	ns	37.33	****	
10	G4	ROS	wl	37.76	****	
18	G6	VIL	dr	41.49	****	
17	G6	VIL	ns	57.39	****	
1	G1	TEX	wl	59.34	****	
13	G5	GOS	wl	66.63	****	

6	G2	NAG	dr	68.34	****	
16	G6	VIL	wl	89.89	****	
5	G2	NAG	ns	90.46	****	
9	G3	SEN	dr	106.04	****	
4	G2	NAG	wl	128.22	****	****
8	G3	SEN	ns	128.48	****	****
7	G3	SEN	wl	251.37		****

Tukey HSD Test; Variable **ARM**; Alpha = 0.050; Error: MQ = 349.34; FG = 36

	No	CV	WSC	**ARM**	1.	2	3
15	G5	GOS	dr	67.90	****		
3	G1	TEX	dr	69.19	****	****	
12	G4	ROS	dr	70.23	****	****	
6	G2	NAG	dr	70.62	****	****	
14	G5	GOS	ns	75.06	****	****	****
9	G3	SEN	dr	79.15	****	****	****
5	G2	NAG	ns	81.12	****	****	****
4	G2	NAG	wl	82.81	****	****	****
2	G1	TEX	ns	87.37	****	****	****
1	G1	TEX	wl	88.22	****	****	****
11	G4	ROS	ns	89.48	****	****	****
13	G5	GOS	wl	89.76	****	****	****
10	G4	ROS	wl	93.79	****	****	****
7	G3	SEN	wl	99.60	****	****	****
8	G3	SEN	ns	100.18	****	****	****
18	G6	VIL	dr	104.66	****	****	****
17	G6	VIL	ns	126.24		****	****
16	G6	VIL	wl	131.42			****

Tukey HSD Test; Variable **Sum-n;** Alpha = 0.050; Error: MQ = 43.000; FG = 36

	No	CV	WSC	**Sum-n** (mean)	1.	2	3	4	5
8	G3	SEN	ns	19.00	****				
9	G3	SEN	dr	19.67	****	****			
7	G3	SEN	wl	22.00	****	****	****		
5	G2	NAG	ns	27.67	****	****	****	****	
6	G2	NAG	dr	29.00	****	****	****	****	
15	G5	GOS	dr	30.00	****	****	****	****	****
17	G6	VIL	ns	30.33	****	****	****	****	****
4	G2	NAG	wl	31.00	****	****	****	****	****
11	G4	ROS	ns	31.67	****	****	****	****	****
10	G4	ROS	wl	32.67	****	****	****	****	****
18	G6	VIL	dr	35.00	****	****	****	****	****
12	G4	ROS	dr	36.00	****	****	****	****	****
13	G5	GOS	wl	38.67	****	****	****	****	****
14	G5	GOS	ns	39.67		****	****	****	****
16	G6	VIL	wl	41.00			****	****	****
3	G1	TEX	dr	42.67				****	****
2	G1	TEX	ns	43.33				****	****
1	G1	TEX	wl	50.00					****

Tukey HSD Test; Variable **Sum-m**; Alpha = 0.050; Error: MQ = 3099E2; FG = 36

	No	CV	WSC	**Sum-m** (mean)	1.	2	3	4	5	6
9	G3	SEN	dr	1556.67	****					
8	G3	SEN	ns	1908.33	****	****				
15	G5	GOS	dr	2003.33	****	****	****			
6	G2	NAG	dr	2010.00	****	****	****			
7	G3	SEN	wl	2111.67	****	****	****	****		
5	G2	NAG	ns	2175.00	****	****	****	****		

4	G2	NAG	wl	2488.33	****	****	****	****		
12	G4	ROS	dr	2520.00	****	****	****	****		
11	G4	ROS	ns	2876.67	****	****	****	****	****	
10	G4	ROS	wl	2918.33	****	****	****	****	****	
3	G1	TEX	dr	2951.67	****	****	****	****	****	
14	G5	GOS	ns	2968.33	****	****	****	****	****	
13	G5	GOS	wl	3468.33		****	****	****	****	
17	G6	VIL	ns	3646.67			****	****	****	
18	G6	VIL	dr	3721.67				****	****	****
2	G1	TEX	ns	3731.67				****	****	****
1	G1	TEX	wl	4373.33					****	****
16	G6	VIL	wl	5238.33						****

Tukey HSD Test; Variable **UR-n**; Alpha = 0.050; Error: MQ = 32.000; FG = 36

	No		WSC	**UR-n** (mean)	1.	2
17	G6	VIL	ns	7.67	****	
1	G1	TEX	wl	7.67	****	
16	G6	VIL	wl	8.00	****	
10	G4	ROS	wl	9.33	****	
7	G3	SEN	wl	9.33	****	
9	G3	SEN	dr	10.33	****	
2	G1	TEX	ns	10.33	****	
11	G4	ROS	ns	11.33	****	
18	G6	VIL	dr	12.33	****	****
8	G3	SEN	ns	14.00	****	****
15	G5	GOS	dr	14.67	****	****
13	G5	GOS	wl	16.00	****	****
14	G5	GOS	ns	16.00	****	****
12	G4	ROS	dr	17.00	****	****
5	G2	NAG	ns	17.00	****	****
3	G1	TEX	dr	17.33	****	****

4	G2	NAG	wl	18.67	****	****
6	G2	NAG	dr	29.33		****

Tukey HSD Test; Variable **UR-m**;Alpha =0050; Error: MQ = 4444.4; FG = 36

No		WSC	**UR-m** (mean)	1.	2
G3	SEN	wl	65.00	****	
G3	SEN	dr	103.33	****	
G4	ROS	wl	105.00	****	
G4	ROS	ns	108.33	****	
G1	TEX	wl	110.00	****	
G1	TEX	ns	111.67	****	
G2	NAG	wl	128.33	****	
G5	GOS	dr	128.33	****	
G6	VIL	wl	135.00	****	
G6	VIL	dr	136.67	****	
G3	SEN	ns	150.00	****	
G6	VIL	ns	153.33	****	
G5	GOS	ns	178.33	****	
G5	GOS	wl	201.67	****	
G1	TEX	dr	208.33	****	
G4	ROS	dr	233.33	****	
G2	NAG	dr	246.67	****	
G2	NAG	ns	265.00	****	

Table S7: Taste thresholds* of compounds **1-14**.

Compound	NR	Mol Weight	Taste Threshold μM/kg
6-Methoxymellein	**1**	208.21	20
Laserinoxid	**2**	406.43	37
2-Epilaserinoxid	**3**	406.43	14
Isovaginatin	**4**	334.46	26
Vaginatin	**5**	334.46	45
Falcarindiol	**6**	260.38	40
Laserin	**7**	390.43	34
Epilaserin	**8**	390.43	20
di-Tig-Germacran	**9**	418.57	8
6-Ang-8-Tig	**10**	418.57	21
6-Tig-8-Ang-Germa	**11**	418.57	47
di-Ang-Germacran	**12**	418.57	27
Falc-3-OAc	**13**	302.41	200
Falcarinol	**14**	244.38	80

*From Schmiech, L.; Uemra, D.; Hofmann, T. Reinvestigation of the bitter compounds in carrots (*Daucus carota* L.) by using a molecular sensory science approach. *J. Agric. Food Chem.* **2008**, *56*, 10252–10260.
